# Chromosome-level genome assembly of *Niphotrichum japonicum* provides new insights into heat stress responses in mosses

**DOI:** 10.3389/fpls.2023.1271357

**Published:** 2023-10-18

**Authors:** Xuping Zhou, Tao Peng, Yuying Zeng, Yuqing Cai, Qin Zuo, Li Zhang, Shanshan Dong, Yang Liu

**Affiliations:** ^1^ Laboratory of Southern Subtropical Plant Diversity, Fairy Lake Botanical Garden, Shenzhen & Chinese Academy of Sciences, Shenzhen, China; ^2^ Colleage of Life Sciences, Guizhou Normal University, Guiyang, China; ^3^ State Key Laboratory of Agricultural Genomics, BGI Research, Shenzhen, China; ^4^ College of Life Sciences, University of Chinese Academy of Sciences, Beijing, China

**Keywords:** moss, genome assembly, heat stress, structural cluster, *Niphotrichum japonicum*

## Abstract

With a diversity of approximately 22,000 species, bryophytes (hornworts, liverworts, and mosses) represent a major and diverse lineage of land plants. Bryophytes can thrive in many extreme environments as they can endure the stresses of drought, heat, and cold. The moss *Niphotrichum japonicum* (Grimmiaceae, Grimmiales) can subsist for extended periods under heat and drought conditions, providing a good candidate for studying the genetic basis underlying such high resilience. Here, we *de novo* assembled the genome of *N. japonicum* using Nanopore long reads combined with Hi-C scaffolding technology to anchor the 191.61 Mb assembly into 14 pseudochromosomes. The genome structure of *N. japonicum*’s autosomes is mostly conserved and highly syntenic, in contrast to the sparse and disordered genes present in its sex chromosome. Comparative genomic analysis revealed the presence of 10,019 genes exclusively in *N. japonicum*. These genes may contribute to the species-specific resilience, as demonstrated by the gene ontology (GO) enrichment. Transcriptome analysis showed that 37.44% (including 3,107 unique genes) of the total annotated genes (26,898) exhibited differential expression as a result of heat-induced stress, and the mechanisms that respond to heat stress are generally conserved across plants. These include the upregulation of *HSP*s, *LEA*s, and reactive oxygen species (ROS) scavenging genes, and the downregulation of *PPR* genes. *N. japonicum* also appears to have distinctive thermal mechanisms, including species-specific expansion and upregulation of the Self-incomp_S1 gene family, functional divergence of duplicated genes, structural clusters of upregulated genes, and expression piggybacking of hub genes. Overall, our study highlights both shared and species-specific heat tolerance strategies in *N. japonicum*, providing valuable insights into the heat tolerance mechanism and the evolution of resilient plants.

## Introduction

Bryophytes (hornworts, liverworts, and mosses) are a group of small, diverse organisms with a long fossil record (Puttick et al., 2018; Harris et al., 2022). They share a haploid-dominant life cycle with un-branched sporophyte growing attached to the gametophyte (Goffinet and Buck, 2013). The phylogenetic relationships among the three bryophyte major lineages and their relationships to other land plants have been contentious for centuries, with distinct scenarios supported by various studies (Cox, 2018; Puttick et al., 2018). With the advent of high-throughput sequencing technology, recent phylogenomic analyses have converged on the hypothesis of a monophyletic bryophyte clade that is sister to the tracheophytes (Puttick et al., 2018; One Thousand Plant Transcriptomes Initiative, 2019; Su et al., 2021). Bryophytes can thrive under the harshest environmental conditions, such as low light intensity, extreme temperatures, low nutrition, and dryness, thus making them the “pioneers” in many ecosystems and providing habitats and conditions for other plants and organisms to live. Bryophytes dominate the terrestrial ecosystem in Antarctica (Ochyra et al., 2008) and can survive in hot deserts [e.g., *Syntrichia caninervis* (Silva et al., 2021)], as they may have evolved an effective stress tolerance genetic toolkit, which may also be related to their distinct morphology and poikilohydric lifestyle (Kulshrestha et al., 2022; Wang et al., 2022). Hence, bryophytes provide excellent models and unique genetic resources for studying stress response and plant resistance traits. However, in contrast to the explosive growth in the number of sequenced angiosperm genomes, resources for bryophyte genomes have accumulated at a slower pace, with only 17 published genomes (**Supplementary Table 1**), in comparison to the 682 publicly available genomes of angiosperms (Sun et al., 2022b), making it difficult to illustrate a comprehensive genetic basis for stress tolerance and to impede a more in-depth comprehension of the genomic evolution of bryophytes.

As previous studies on environmental stress of bryophytes mainly focused on drought tolerance (Wang et al., 2022), the molecular mechanisms of bryophytes responding to other stresses, such as heat, are largely unknown. Studies in liverwort *Marchantia polymorpha* indicated that the core components of the heat stress response are conserved between bryophytes and angiosperms (Marchetti et al., 2021; Tan et al., 2023). During heat stress, the genes encoding the cyclic nucleotide gated calcium channels (*CNGC*s) are activated (Finka et al., 2012; Finka and Goloubinoff, 2014), leading to the opening of heat-sensitive calcium-permeable channels in plants, causing an inward Ca^2+^ flux and initiating a signal transduction network that regulates the expression of a number of genes, including the heat shock transcription factors (*HSF*s), heat shock proteins (*HSP*s), and reactive oxygen species (ROS) scavenging enzymes, to enhance heat tolerance (Zhu, 2016; Marchetti et al., 2021). Global warming has significantly impacted numerous natural ecosystems, exacerbating natural disasters such as extreme heat and causing devastating damage to crop production (Lobell et al., 2011; Lesk et al., 2016). Without effective adaptation and genetic improvement, such damage to crops (wheat, rice, maize, and soybean) is expected to increase (Zhao et al., 2017). Hence, there is an urgent need to identify and characterize heat-resistant genetic resources and to facilitate the improvement of heat tolerance in crops.

*Niphotrichum japonicum* (Grimmiaceae, Grimmiales) is a vigorous moss species that usually grows on dry rocks exposed to intense light (**Supplementary Figure 1**). As an important horticultural moss, this species has been extensively utilized to green roofs and walls of urban buildings due to its impressive ability to withstand environmental stresses (heat, drought, high light, and nutrient limitation) (Akita et al., 2011; Hendrawan and Murase, 2011). Several studies have investigated the strontium stress (Ren et al., 2023), cold, heat, and drought tolerance (Lei et al., 2021; Xia et al., 2022) of *N. japonicum* from the perspective of photosynthetic regulation. However, the genetic level of stress tolerance in *N. japonicum* remains largely unknown due to the absence of a high-quality reference genome. In this study, we present a chromosome-level genome assembly for *N. japonicum*. Through comparative genomic and transcriptomic studies, we have uncovered conserved elements of the heat response process in *N. japonicum*, identified unique genes that may play a role in the species’ heat resistance, and revealed the heat-responsive genes, modules, and structural clusters in the genome. Our study not only illuminates the heat response of the heat-tolerant moss, but also offers significant genetic resources for future research on embryophyte evolution, gene function, and stress response in land plants.

## Results

### A chromosome-level genome assembly

**Figure 1:**
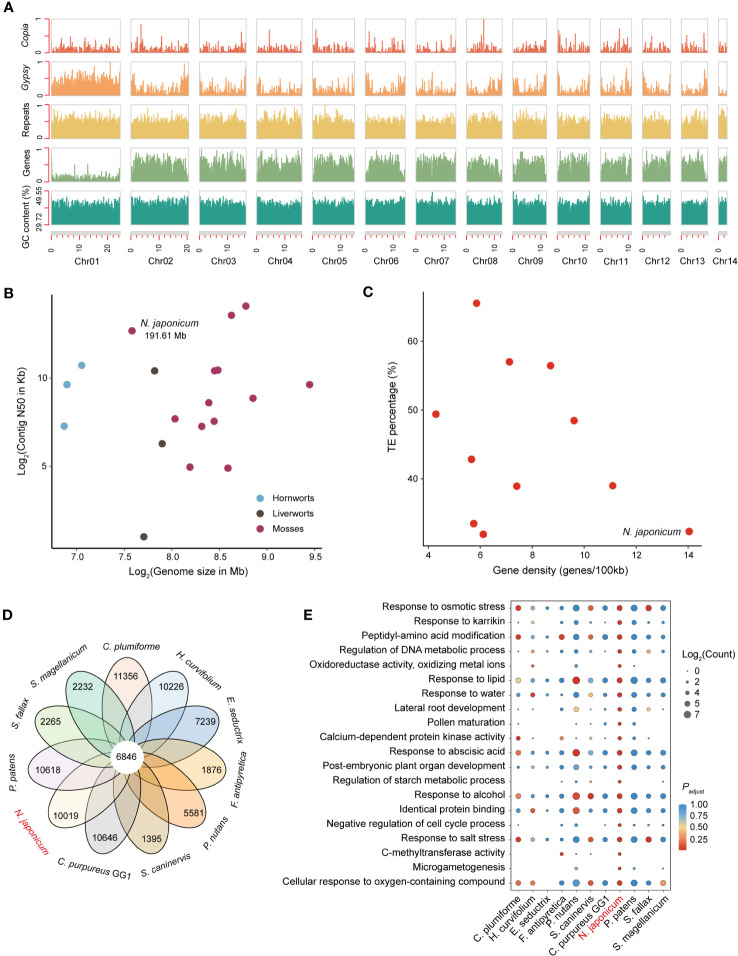
Genomic characterization and comparative genomic analysis of *N. japonicum*. **(A)** Density plots across *N. japonicum* chromosomes (in Mb), showing the number or proportion (GC content) of 100-Kb sliding window with 90-Kb jump of each feature. The number of the features is normalized (0,1) except for the GC content. **(B)** Comparison of genome size and contig N50 among 18 assembled bryophyte genomes. **(C)** Comparison of gene density and TE percentage among 11 assembled moss genomes. **(D)** Petal diagram analyses showing shared and unique genes in 11 mosses. **(E)** GO functional enrichment of unique genes in 11 mosses. All unique genes of the 11 mosses annotated by the GO library are used as background information, with *N. japonicum* as the focal species, and only the GO terms in the top 20 of the adjusted *p*-value (*p*_adjust_) are shown.

Here, we reported a high-quality, chromosome-level genome assembly of *N. japonicum* gametophyte based on a combination of 120.08 Gb Illumina short reads, 79.16 Gb Nanopore long reads, and 116.88 Gb Hi-C data. Based on *K*-mer frequency distribution of 115.24 Gb clean Illumina short-read data, the genome size was estimated as 184.22 Mb (**Supplementary Figure 2**). After conducting assembly and removing contamination (**Supplementary Figure 3**), we obtained an optimized assembly of 191.61 Mb with a contig N50 length of 6.60 Mb and a scaffold N50 length of 14.23 Mb, corresponding to the 14 chromosomal pseudomolecules (**Table 1**, **Figure 1A**, and **Supplementary Figure 4**). The *N. japonicum* genome assembled was the smallest among all moss genomes assembled to date, and the length of contig N50 ranked third, demonstrating its high genome continuity (**Figure 1B**). Repeat sequences of *N. japonicum* constituted 34.74% of the genome with transposable elements (TEs) being the predominant component by accounting for 32.34% of the genome, and long terminal repeat sequences (LTRs) being the major component within TEs (**Table 1** and **Supplementary Table 2**). The genome coded for 26,898 protein-coding genes (PCGs), and was compact, having the highest gene density among the published bryophytes (**Table 1** and **Figure 1C**). Approximately 82.27% of the PCGs were functionally annotated using InterProScan software, the gene ontology (GO), and Kyoto Encyclopedia of Genes and Genomes (KEGG), Swiss-Prot, TrEMBL, and the *Arabidopsis* Information Resource (TAIR) databases (**Supplementary Table 3**). Based on the Benchmarking Universal Single-Copy Ortholog (BUSCO) estimation using the Viridiplantae odb10 dataset, the completeness of the gene space captured by the *N. japonicum* genome was ca. 97.00%, which was comparable to the model moss *Physcomitrium patens* v3.3 with a BUSCO score of 98.84% (**Supplementary Figure 5**). Moreover, approximately 88.79% of the annotated genes of *N. japonicum* were captured in the transcriptome unigenes.

**Table 1:** Assembly and annotation statistics of the *N. japonicum* genome.

Assembly	
Genome size (bp)	191,608,406
Longest scaffold (bp)	24,499,556
N50 of scaffold (bp)	14,234,889
Longest contig (bp)	15,025,146
N50 of contig (bp)	6,604,031
GC ratio (%)	41.86
Annotation	
Number of protein-coding genes	26,898
Gene density (genes/100 kb)	14.39
Repeats in genome (%)	34.74
Transposable elements in genome (%)	32.34

In the Hi-C heatmap of *N. japonicum* (**Supplementary Figure 4**), Chr01 was the largest chromosome (24.50 Mb) and showed few contacts with the other chromosomes. Furthermore, Chr01 had the lowest gene density (33 genes/Mb) and the highest repeat proportion (70.81%) among all chromosomes (**Figure 1A**), indicating it as a putative sex chromosome (containing 807 genes).

### Comparative genomic analysis

Except for WGD, all the gene duplicated modes were categorized as single-gene duplication (Freeling, 2009), consisting of tandem duplicates (TD), proximal duplicates (PD), transposed duplicates (TRD), and dispersed duplicates (DSD) (Qiao et al., 2019). Our identification of duplicated genes on the sex chromosomes indicated that genes located on these chromosomes in mosses were highly variable, with numerous genes duplicated. The highest percentage of duplicated genes occurred in *Sphagnum magellanicumn* (48.33%), followed by *N. japonicum* (36.80%), with an average of approximately 23.92% (**Supplementary Figure 6**). Moreover, our results demonstrated the lack of WGD genes in the moss sex chromosomes.

To identify the unique genes of *N. japonicum*, we clustered the proteomes of *N. japonicum* with those of the other 10 published moss genomes (i.e., *S. magellanicum*, *Sphagnum fallax*, *P. patens*, *S. caninervis*, *Ceratodon purpureus*, *Pohlia nutans*, *Fontinalis antipyretica*, *Entodon seductrix*, *Hypnum curvifolium*, and *Calohypnum plumiforme*) (**Supplementary Table 1**). A total of 6,846 genes were shared across 11 mosses, and 10,019 unique genes were identified for *N. japonicum* (**Figure 1D**). GO enrichment demonstrated that *N. japonicum* unique genes were significantly enriched in “response to osmotic stress”, “response to karrikin”, and “oxidoreductase activity, oxidizing metal ions” (**Figure 1E** and **Supplementary Table 4**), comply with the species-specific competences in withstanding stress and in post-stress germination (Inupakutika et al., 2016; Zheng et al., 2020; Sepulveda et al., 2022).

We identified a total of 729 transcription factors (TFs) in 62 TF families for the *N. japonicum* genome, in contrast to the 1,185 TFs of the *P. patens* genome (**Supplementary Table 5**). A majority of the TF families had a ratio of nearly 1:2 in number between *N. japonicum* and *P. patens* (**Supplementary Table 5**), possibly caused by the fact that *P. patens* had undergone an additional lineage-specific WGD (Lang et al., 2018). We also found that four TFs (B3, HB-WOX, LIM, and SRS) present in higher numbers in *N. japonicum* than in *P. patens*, particularly the B3 TF (**Supplementary Table 5**) that mediated desiccation tolerance in plants (Carbonero et al., 2017), suggesting possibly a reinforced desiccation tolerance trait in *N. japonicum*.

### Whole-genome duplication and inter-genomic synteny

**Figure 2:**
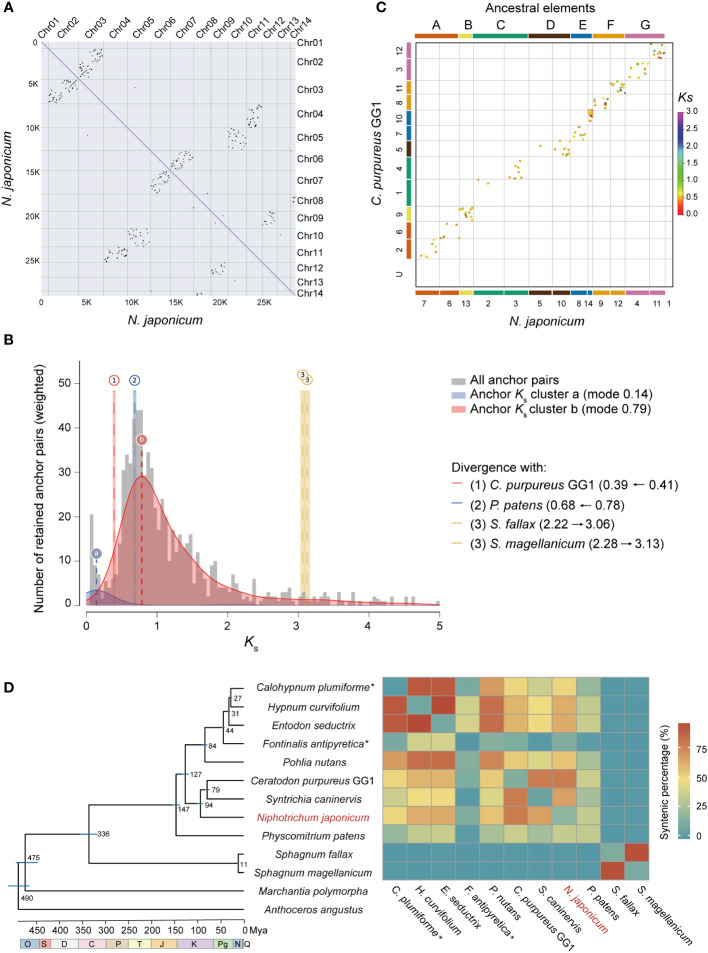
WGD analysis of *N. japonicum* and inter-genomic synteny of mosses. **(A)** Intra-genomic synteny of *N. japonicum*. **(B)** Substitution-rate-adjusted mixed paralog–ortholog *K*_s_ plot for *N. japonicum*. The anchor-pair *K*_s_ distribution for *N. japonicum* is shown in gray, with two *K*_s_ peaks inferred by lognormal mixture model clustering shown in blue and red. The vertical dashed lines labeled “a” and “b” indicate the modes of these components and *K*_s_. Divergence events between *N. japonicum* and other species are represented by long vertical dashed lines but by Arabic numerals, with the left and right ranges of the colored rectangles representing standard deviations (SD). Identical divergence events are indicated by the same color and number, i.e., event 3 in this study. The numbers on the right represent the *K*_s_ of the corresponding event, and the horizontal arrows represent the *K*_s_ change of the species divergence events resulting from the substitution-rate-adjusted. **(C)** Inter-genomic synteny of *N. japonicum* and *C purpureus* GG1; the colors of the two genome chromosomes correspond to the ancestral elements, indicating that the extant chromosomes are duplicated from different ancestral elements. **(D)** Chronogram and synteny of mosses based on whole-genome data. All branches are maximally supported by bootstrap values (ML). O, Ordovician; S, Silurian; D, Devonian; C, Carboniferous; P, Permian; T, Triassic; J, Jurassic; K, Cretaceous; Pg, Paleogene; N, Neogene; Q, Quaternary. The times of speciation are marked on the branches, and the range of blue bars indicates the 95% confidence interval of the divergence time. *, non-chromosomal level genome assembly. Mya, million years ago. The heatmap on the right represents synteny of intra- or inter-genomes.

Based on the analysis of intra-genomic synteny in *N. japonicum*, we identified 157 syntenic blocks, containing 1,823 genes, accounting for 6.75% of the genome (**Figure 2A**). We additionally identified the whole-genome duplication (WGD) event using the distribution of the substitution-rate-adjusted number of substitutions per synonymous site (*K*_s_). The *K*_s_ distribution showed two peaks (i.e., “a” and “b”) (**Figure 2B**). The *K*_s_ value of “a” was very small in both *K*_s_ unit (0.14) and *K*_s_ height, with only a few retained duplicated genes (i.e., less than five anchor pairs). Therefore, we inferred that “a” was generated by single gene duplication events rather than WGD. The *K*_s_ peak of the WGD event in *N. japonicum* was at 0.79 (**Figure 2B**), which was larger than the divergence peak of *N. japonicum* with *P. patens* (0.68), indicating that the WGD event in *N. japonicum* occurred before the divergence with *P. patens* and that they shared a common WGD event, i.e., the “ψ” event (Gao et al., 2022). Furthermore, the abundant synteny between the *C. purpureus* GG1 and *N. japonicum* chromosome indicated the presence of the seven ancestral chromosomal elements of *N. japonicum* (**Figure 2C**).

To investigate the synteny between the genome assembly of *N. japonicum* and the published moss genomes, we identified syntenic gene pairs using the jcvi software (Tang et al., 2008). The *N. japonicum* genome had varying degrees of synteny with other moss genomes, depending on the phylogenetic distance, with the exception of *Sphagnum* (**Figure 2D**).

### Characterization and function divergence of duplicated genes

**Figure 3:**
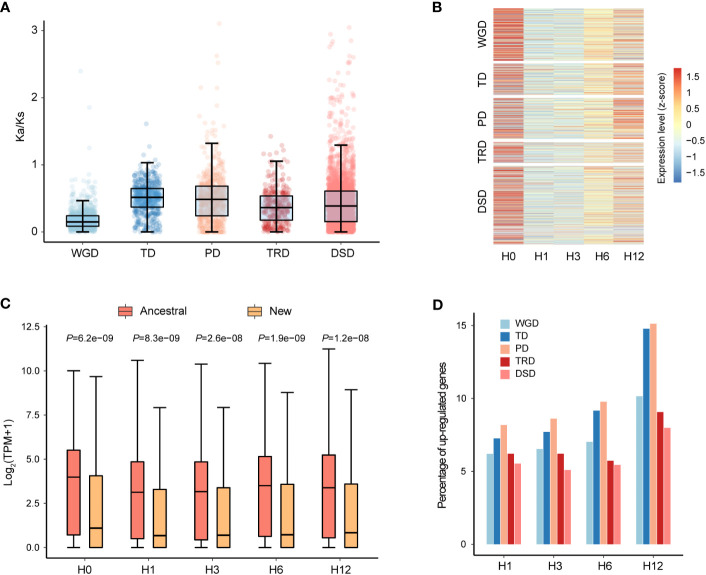
Gene characteristics of five different duplication modes in *N. japonicum*. **(A)** Box plots showing the *K*_a_/*K*_s_ ratio of gene pairs derived from different modes of duplication. **(B)** Expression patterns of duplicated genes of five modes. H0 represents the control (20°C) and H1, H3, H6, and H12 represent heat stress at 42°C for 1, 3, 6 and 12 h, respectively. **(C)** Comparison of the expression levels of ancestral and new TRD genes. *p*-values were calculated using the Wilcoxon test. **(D)** Heat stress response of different modes of duplicated genes in *N. japonicum*.

*N. japonicum* possessed 7,835 duplicated genes, representing 29.13% of the 26,898 genes, including 1,823 WGD genes, 1,103 TD genes, 1,382 PD genes, 464 TRD genes, and 3,063 DSD genes. The *K*_a_ (number of substitutions per nonsynonymous site), *K*_s_, and *K*_a_/*K*_s_ values were estimated for gene pairs generated by different modes of duplication. The *K*_a_/*K*_s_ values among different modes of gene duplications showed a striking trend, with TD and PD genes having higher *K*_a_/*K*_s_ values than other duplication modes, while WGD genes had the lowest *K*_a_/*K*_s_ value (**Figure 3A** and **Supplementary Table 6**). This finding suggested that TD and PD genes played crucial roles in evolving new functions in *N. japonicum*.

We carried out further investigation into the expression patterns of duplicated genes of different categories under heat stress. The expression levels were examined for genes of WGD, TD, TRD, and DSD, and it was observed that the majority of these genes had the highest expression levels at 0 h (H0) of heat treatment, which was the lowest at 1 h (H1) and 3 h (H3) (**Figure 3B**). On the contrary, for PD genes, a greater number of genes expressed the highest levels at 12 h (H12) of treatment (**Figure 3B**). Interestingly, the ancestral gene locus in the TRD gene pairs showed significantly higher expression levels (*p* < 0.001) than the new gene locus during all periods of heat stress (**Figure 3C**). This expression divergence could be attributed to their different responses to the environment or functional redundancy, implying the possibility of sub-functionalization, neo-functionalization, or pseudogenization after gene duplication occurred.

According to our identification of differentially expressed genes (DEGs) in duplicated genes, a total of 1,043 (13.31% of all) duplicated genes (249 WGD genes, 185 TD genes, 246 PD genes, 55 TRD genes, and 308 DSD genes) were upregulated under heat stress, including 328 unique genes (**Supplementary Table 7**). Categories of PD and TD had a higher proportion of genes upregulated, which increased with the duration of treatment, suggesting the potential role in high-temperature tolerance (**Figure 3D**). The GO enrichment analysis of the upregulated genes in each duplicated category under heat stress demonstrated differing functional profiles among genes from various duplicated types (**Supplementary Figure 7** and**Supplementary Table 8**). For instance, PD genes predominantly functioned in the heat stress response with the term “regulation of response to oxidative stress” being specific to PD. TRD boasted more distinct gene functions in comparison to other modes of duplication, mainly related to the transmembrane transport of carbohydrates.

### Expression pattern of key gene families related to heat stress

Overall, we identified 10,070 DEGs (differentially expressed in one or more conditions), including 3,107 unique genes. Of these, 3,819 were upregulated (including 92 TFs and 1,606 unique genes) in one or more of the treated samples versus the control samples and 6,253 were downregulated (including 283 TFs and 1,501 unique genes) (**Supplementary Table 9**), with the strongest response observed after 12 h of heat treatment (**Supplementary Figure 8**). The upregulated genes were enriched in the GO terms closely related to heat stress, such as “protein folding”, “response to heat”, “response to reactive oxygen species”, and “response to hydrogen peroxide” at all stages (**Supplementary Figure 9** and **Supplementary Table 10**). The downregulated genes were enriched in a number of GO terms related to “photosynthesis”, “ATP synthesis”, “cell wall remodeling”, “transmembrane transport”, and “response to fungus” (**Supplementary Figure 10** and **Supplementary Table 10**), suggesting that the energy metabolism and microbial resistance pathways in *N. japonicum* were strongly inhibited or inactivated under heat stress.

The plant self-incompatible protein S1 (Self-incomp_S1) family (PF05938) or self-incompatible protein homologs (SPHs) were established on the basis of homology to *PrsS* gene and may be associated with programmed cell death (Ride et al., 1999; Rajasekar et al., 2019). We scanned the genomes of algae (chlorophytes and charaphytes), bryophytes (hornworts, liverworts, and mosses), and tracheophytes (lycophytes, ferns, gymnosperms, and angiosperms), and found the largest number of *Self-incomp_S1*s is in *Arabidopsis thaliana* (65), followed by *N. japonicum* (56), whereas members of this family were not found in algae, hornworts, ferns, and gymnosperms, suggesting a conspicuous expansion of Self-incomp_S1 family in *N. japonicum* (**Supplementary Table 11**). Differential expression analysis revealed that 15 members of Self-incomp_S1 family (12 upregulated and 3 downregulated in one or more conditions) were significantly induced (*p* < 0.05) by heat treatment of *N. japonicum* at 42°C, among which, *NJ13G007760* accumulated more transcripts and had log_2_ (fold change (FC)) > 6 (**Supplementary Table 9**).

**Figure 4:**
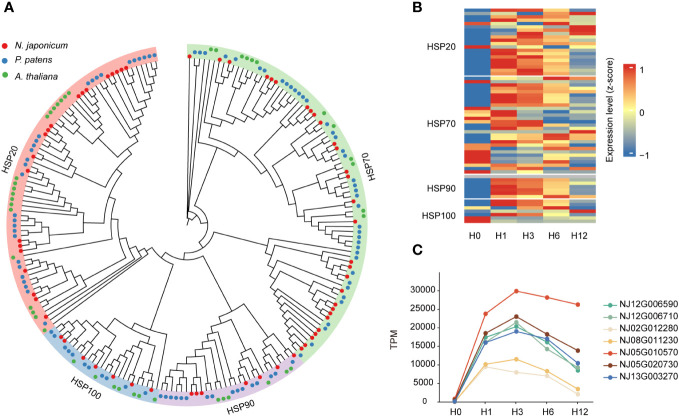
Phylogeny of *HSP*s and their expression levels under heat stresses. **(A)** Phylogeny of the *HSP*s. **(B)** Heatmap showing the gene expression levels of *HSP*s under control condition and 42°C heat stresses (at 1, 3, 6, and 12 h) in *N. japonicum*. **(C)** Differential expression levels of seven *HSP20* members under heat stress.

Building upon a previous study conducted in *A. thaliana* (Swindell et al., 2007), we identified 63 HSPs from the *N. japonicum* genome, including four subfamilies, namely, HSP20 or sHSP (20), HSP70 (29), HSP90 (7), and HSP100 (7) (**Figure 4A**). Forty *HSP* genes were upregulated in one or more of the treated samples versus the control samples (**Supplementary Table 12**). We further analyzed the expression patterns of *HSP* genes at different stages. At H0, the majority of *HSP*s were expressed at low levels; after heat exposure, the expression levels of *HSP*s increased, more obviously at the H1 and H3 stages, and slightly decreased at the stages of H6 and H12 (**Figure 4B**, and **Supplementary Table 12**). Among them, the expression level of the HSP20 subfamily was higher than that of the other subfamilies at all stages of stress, and an examination of *HSP*s in *P. patens* also yielded similar results (**Supplementary Figure 11** and **Supplementary Table 12**). Further analysis of the HSP20 subfamily revealed that the TPM (transcript per million) values of seven genes were all higher than 10,000 at the H1 and H3 stages, and differential expression analysis showed that their log_2_ (FC) was >5, and the total expression level (TPM) under stress (H1, H3, H6, and H12) was 3.36 times higher than that of the other 56 *HSP*s (**Figure 4C** and **Supplementary Table 12**); in addition, one *HSP70* gene (*NJ09G004040*) was significantly upregulated at stage H12 (*p* < 0.001), with a TPM value reaching 642 at the H12 stage in contrast to TPM being 0 at other stages (**Supplementary Table 12**).

A total of 41 Late embryogenesis abundant (*LEA*) genes were identified in *N. japonicum*, compared with 35 in *P. patens* (**Supplementary Figure 12** and **Supplementary Table 13**). The number of *LEA* genes varied widely among 11 mosses (from 29 to 70) (**Supplementary Table 13**). *N. japonicum* had the largest dehydrin (DHN) subfamilies and a total number of *LEA* genes similar to that of the desert moss *S. caninervis* (40), which could be related to its potential drought resistance. We further analyzed the expression patterns of *LEA* genes in *N. japonicum* under heat, and differential expression analysis showed that 11 *LEA* genes were upregulated under heat stress (**Supplementary Table 9**), among which three genes, *viz*., *NJ08G014280* (*LEA_2*), *NJ03G019430* (*LEA_4*), and *NJ05G015930* (*DHN*), had TPM > 1,000 and log2(FC) > 1.

We identified a total of 92 pentatricopeptide repeat (*PPR*) genes in the *N. japonicum* genome (**Supplementary Table 14**). Under heat stress, only seven *PPR* genes were upregulated in one or more conditions, whereas 26 *PPR* genes were downregulated (**Supplementary Table 14**), which could be due to heat-induced disintegration or functional inhibition of *N. japonicum* chloroplast. We also found that the expression of 16 chlorophyll A–B binding proteins, which acted as photochemical reaction centers in light absorption, was decreased in one or more conditions under heat stress (**Supplementary Table 14**), suggesting that chlorophyll degradation occurs under heat treatment.

We searched the *N. japonicum* genome for genes associated with ROS scavenging, based on previous studies in *A. thaliana* (Inupakutika et al., 2016). One gene (*NJ08G015430*) encoding blue copper protein, one gene (*NJ04G021770*) encoding glutathione S-transferase, one gene (*NJ07G010200*) encoding ferritin, one gene (*NJ07G019160*) encoding glutathione peroxidase, and seven genes (*NJ02G021000*, *NJ04G009910*, *NJ07G001270*, *NJ07G001310*, *NJ07G023040*, *NJ10G011570*, and *NJ11G007530*) encoding thioredoxin were significantly upregulated (*p* < 0.05) in response to heat stress and could play a role in scavenging excess ROS (**Supplementary Table 9**).

### Profiling the heat stress response in *N. japonicum*

As some upregulated genes tended to accumulate transcripts in the form of physical clusters, e.g., *NJ02G020390*, *NJ02G020400*, *NJ02G020410*, *NJ02G020420*, *NJ02G020430*, and *NJ02G020440* (**Supplementary Table 9**), the workflow of Pecrix et al. (2018) was used to identify physical clusters of co-localized and co-regulated genes or islands. Our results showed that the number of islands ranged from 27 to 29 and contained 116 to 360 genes across the four stages of heat stress, with the largest island being ~30 Kb (**Table 2** and **Supplementary Table 15**). In addition, we found that the mean and median log_2_ (FC) values of the island genes appeared to show a gradual decrease from heat stress stages of H1 to H12 (**Table 2**). Given the short physical distance in TD and PD gene pairs, we searched for duplicated genes in such islands, indicating that islands of H1UP, H3UP, H6UP, and H12UP contained 18.52%, 20.97%, 22.41%, and 20.56% of duplicated genes, respectively (**Table 2** and **Supplementary Table 15**). This suggested that gene duplication was not the only explanation for gene organization in islands, which resembled the pattern seen for the symbiosis-related gene islands in *Medicago truncatula* (Pecrix et al., 2018).

**Table 2:** Features of islands in upregulated genes of *N. japonicum* under heat stress.

Patterns	Total number of genes^a^ with relevant pattern^b^ on the chromosomes	Number of genes with relevant pattern^b^ in islands	Number of islands	Mean island size^c^ (bp)	Mean log_2_(FC)^c^ of island genes (median)	Mean number^c^ of genes with relevant pattern^b^ per island (max)	Number of duplicated genes with relevant pattern^b^ in islands (percentage)
H1UP	1,914	135	29	23,458 ± 2,422	3.51 ± 0.22 (2.47)	4.66 ± 0.37 (10)	25 (18.52%)
H3UP	1,900	124	27	24,502 ± 2,181	3.20 ± 0.22 (2.32)	4.59 ± 0.38 (11)	26 (20.97%)
H6UP	2,002	116	27	24,567 ± 2,608	3.04 ± 0.22 (2.04)	4.30 ± 0.26 (7)	26 (22.41%)
H12UP	3,032	360	74	29,881 ± 2,067	2.65 ± 0.12 (1.78)	4.86 ± 0.26 (15)	74 (20.56%)

**^a^** Nuclear genes on the chromosomes, whether in islands or not. **^b^** Include H1UP (upregulated in H1 samples versus H0 samples), H3UP (upregulated in H3 samples versus H0 samples), H3UP (upregulated in H6 samples versus H0 samples), and H12UP (upregulated in H12 samples versus H0 samples). **^c^** ± Standard error of mean (SEM).

As for the biological function of PCGs in these islands, in addition to HSPs, profilin and some proteins of unknown function are also present as physical clusters in different islands (**Supplementary Table 15**). Profilin plays a role in cell elongation, cell shape maintenance, and the determination of flowering time in *A. thaliana* (Ramachandran et al., 2000). A number of other important genes were also identified in these islands, such as the mechanosensitive ion channel family, which could be associated with early signaling of heat stress, the MYB TF associated with plant adversity, and the ABC transporter (**Supplementary Table 15**).

**Figure 5:**
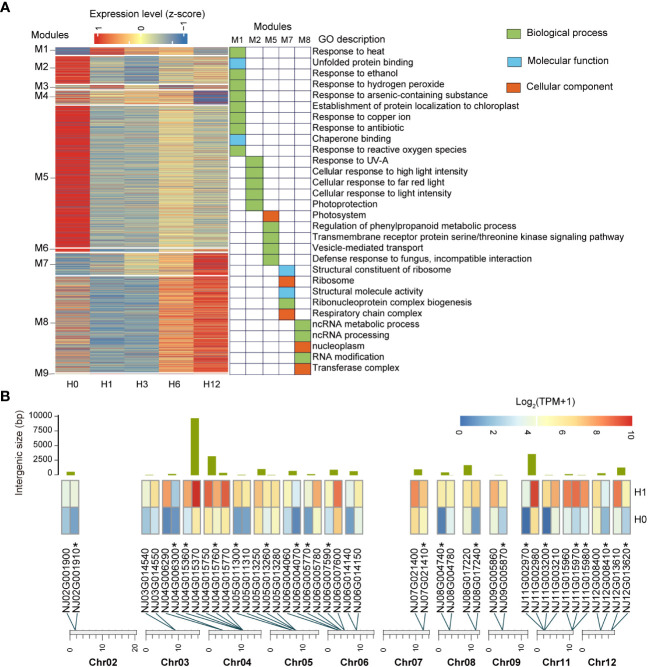
Characteristics of *N. japonicum* in response to heat stress. **(A)** Heatmap showing the relative expression levels of genes in nine co-expression modules by WGCNA across five stages of the heat stress. GO enrichment analysis was performed on genes from modules that showed a high correlation at one specific stage, all genes of *N. japonicum* annotated by GO library as background information, and the *p*_adjust_ < 0.05 for 30 displayed GO terms. **(B)** Distribution and expression patterns of neighboring genes in hub genes. The bars represent the intergenic spacer length of the neighboring genes. Heatmap showing the relative expression levels of neighboring genes at H0 and H1 stages. *, unique genes.

To better understand the dynamic changes in gene regulation and regulatory programs during heat stress, we performed a weighted correlation network analysis (WGCNA) and identified nine co-expression modules at different stages of the *N. japonicum* under heat stress (**Figure 5A**). The number of genes in the modules ranged from 24 (M9) to 8,018 (M5). The modules are enriched in the GO functional terms of photoprotection, defense against micro-organisms and nutrient metabolism (M2 and M5), removal of toxic substances and protein homeostasis maintenance (M1), and protein biosynthesis (M7 and M8) (**Figure 5A** and **Supplementary Table 16**). We observed a significant (*p* < 0.05) enrichment of GO terms associated with antibiotics.

From M1, we identified 122 putative hub genes that were significantly associated with H1(*p* < 0.05), based on module membership (MM) ≥ 0.6 and gene significance (GS) ≥ 0.5 (**Supplementary Table 17**). The members of many (80) of these genes had been identified as homologs in *P. patens* or *A. thaliana*. Besides *HSF*s and *HSP*s, which had a dominant function in heat stress, vital genes such as *ORP1B*, *HBP2*, *BAG6*, and *ROF2* were found. There were also numerous unique genes (41) that they could represent unique and novel players in *N. japonicum* heat stress.

Close examination revealed that some of the hub genes were located adjacent to each other on the chromosome (20 groups containing 43 genes) and that they were not duplicated genes, all of which had upregulated TPM levels under heat stress, typically each group contained two to three genes and the physical distance between neighboring genes was 18–9,738 bp (average ~1,166 bp) (**Figure 5B** and **Supplementary Table 17**).

## Discussion

### Structural evolution of the crown group mosses

Based on a high-quality, chromosome-level genome assembly of *N. japonicum*, we investigated the structural evolution of available moss genomes using syntenic analyses. The autosomes of mosses are generally highly syntenic depending on the phylogenetic distance as has been reported by Yu et al. (2022). The lack of synteny between *Sphagnum* and other mosses may be due to the very early divergence of it from other extant mosses (Healey et al., 2023). The Hi-C contact characteristic of *N. japonicum* sex chromosome, gene density, and repeat density of the *N. japonicum* sex chromosome conform to those previously observed in *Sphagnum* (Healey et al., 2023), *C. purpureus* (Carey et al., 2021), and *E. seductrix* and *H. curvifolium* (Yu et al., 2022). However, we find that sex chromosome length varies significantly among mosses. *N. japonicum*, *S. caninervis* (Silva et al., 2021), and *C. purpureus* (Carey et al., 2021) have the largest sex chromosomes in their genomes, whereas the two *Sphagnum* species (Healey et al., 2023) and the two Hypnales (Yu et al., 2022) possess the smallest.

Gene duplication provides genetic material for evolutionary innovation and is considered as an important driver for diversification and evolution (Van de Peer et al., 2017). Throughout the evolutionary history of land plants, there have been multiple occurrences of ancestral WGD events (Van de Peer et al., 2017). The genome of *N. japonicum* largely retained the feature of the seven ancestral chromosomes, similar to that of *P. patens* (Lang et al., 2018) and *C. purpureus* GG1 (Carey et al., 2021), but different from the five ancestral chromosomes of *Sphagnum* (Healey et al., 2023). In addition, it is noteworthy that Chr01 lacks synteny with autosomes (**Figure 2A**) or with *C. purpureus* (**Figure 2B**), suggesting a unique evolutionary mechanism. Considering the 1:2 syntenic relationship of the ancestral chromosomes of mosses with those of *N. japonicum*, the absence of collinearity between Chr13 and other autosomes may indicate that the sex chromosome of *N. japonicum* originated from the other copy of the ancestral chromosome B (**Figures 2A, B**), albeit with disrupted gene order (Carey et al., 2021). This could also clarify why the WGD gene is absent from the sex chromosome.

It is commonly acknowledged that the lack of meiotic recombination reduces the effectiveness of natural selection, leading to degradation and gene loss on non-recombinant chromosomes (e.g., mammalian Y chromosome and other UV systems) that typically contain, at best, orders of magnitude fewer genes (Charlesworth et al., 2000; Ferris et al., 2010; Bachtrog, 2013; Ahmed et al., 2014; Iwasaki et al., 2021). However, this is not the case in *N. japonicum* as well as other mosses, which typically contain hundreds to thousands of genes on their sex chromosomes (Carey et al., 2021; Silva et al., 2021; Yu et al., 2022; Healey et al., 2023). Our analyses of gene duplicated modes show that a significant proportion of genes on these sex chromosomes exhibit single-gene duplication, indicating that single-gene duplication may be beneficial in preventing the loss of genes on sex chromosomes of mosses due to the suppressing of recombination (Carey et al., 2021).

### Conserved elements of heat stress response in plants

HSPs are important functional proteins that are induced by heat and are known to be targeted by heat-associated TFs. Under heat stress, HSPs can act as molecular chaperones, binding with other proteins and playing a crucial role in regulating protein quality by renaturing various proteins that have been denatured by heat stress (Kotak et al., 2007; Ohama et al., 2017; Waters and Vierling, 2020). HSPs of *N. japonicum* and *P. patens* were identified, and a putative 1:2 ratio between the two species would suggest either more loss of HSPs in *P. patens* or more retention of HSPs in *N. japonicum*. Notably, 63% of *HSP*s were upregulated in one or more conditions, which was associated with the high-temperature response of *N. japonicum* (**Supplementary Table 12**), a response that appears to be highly conserved in animals, yeast, and prokaryotes (Finka et al., 2011). Furthermore, we found a strong upregulation of *HSP20*, which may play a role in protecting the stability of the *N. japonicum* membrane system (Rütgers et al., 2017; Arena et al., 2019).

LEAs were small, heat-stable, hydrophilic proteins that are synthesized in orthodox seeds during mid to late maturation and may be associated with tolerance to abiotic stresses, such as drought, salinity, and high or cold temperature (Delahaie et al., 2013; Xu et al., 2018; Chen et al., 2019; Liu et al., 2019a; Zhuo et al., 2020). *N. japonicum* has 41 *LEA*s, which is higher than *P. paten*s (35), but is not an expansion (*P. nutans* and *C. purpureus* have 70 and 62, respectively) (**Supplementary Table 13**). The *LEA* genes may play a crucial role in the high-temperature stress response of mosses *Bryum argenteum* (Zhuo et al., 2020) and *S. caninervis* (Silva et al., 2021). Our results (11 *LEA*s upregulated in one or more conditions) also provide further evidence for their potential role under heat stress (**Supplementary Table 9**).

The PPR gene family in *N. japonicum* appears to be contracted compared to *Anthoceros angustus* (Zhang et al., 2020) and *P. patens* (Xu et al., 2018). The *PPR* genes are mainly located in mitochondria or chloroplasts, which may be closely related to RNA editing sites and plant growth and development (Guillaumot et al., 2017; Zhang et al., 2020). Under high temperature stress, chloroplasts undergo extensive proteomic remodeling, and the efficiency of nuclear-encoded precursor proteins to translocate to chloroplasts is inhibited (Zheng et al., 2022). It is therefore not surprising that the function of the *PPR* genes is repressed.

ROS is a key signaling molecule that regulates many biological processes (Mittler, 2017). However, under heat stress, plants accumulate a large amount of ROS, including _1_O^2^, O_2_^−^, H_2_O_2_, and OH^−^, which can cause oxidative damage to plant cells (Kissen et al., 2016; Yin et al., 2018). ROS homeostasis is critical for plant resistance and adaptation to environmental stresses (Miller, 2012). We identified ROS scavenging genes within the *N. japonicum* genome. Eleven of these genes were upregulated during high-temperature stress, indicating that they may play a role in mitigating these conditions. This process appears to be highly conserved during plant response to high-temperature stress (Li et al., 2018a).

### Innovations of heat stress response in *N. japonicum*

Among the five models of gene duplication, TD and PD showed higher *K*_a_/*K*_s_ values. This suggests that, like other plant lineages, they may have undergone faster sequence divergence (Qiao et al., 2019). In *A. thaliana*, TD genes play unique roles related to “binding” and “activity”, whereas PD genes are linked to apoptosis and immune responses (Qiao et al., 2019). Functional enrichment of upregulated genes under heat stress in *N. japonicum* indicates functional differences between various gene duplication models (**Supplementary Figure 7** and **Supplementary Table 8**). For instance, PD may have a unique scavenging excess ROS role (Kerchev and Van Breusegem, 2022), while TRD genes may be involved in coordinating tolerance to oxidative and osmotic stress in response to heat stress (Saddhe et al., 2021).

Although most of the gene families associated with plant resistance did not undergo an expansion in *N. japonicum* (compared with other bryophyte genomes), the gene family encoding plant self-incompatibility protein S1 did show distinct expansions (**Supplementary Table 11**). This family is confined to tracheophytes and mosses (**Supplementary Table 11**), and consists of a series of plant proteins that are related to the *Papaver rhoeas* self-incompatibility protein S1 (*PrsS*). *PrsS* is a self-incompatibility determinant (Foote et al., 1994) and can be ectopically expressed, inducing growth arrest and cell death of vegetative cells independent of the reproductive context (Lin et al., 2020). The expansion of the plant Self-incomp_S1s in *N. japonicum* and their differential expression responding to heat stress might suggest their role in regulation of growth and stress responses (Rajasekar et al., 2019; Lin et al., 2020).

We identified a large number of DEGs in the transcriptome of *N. japonicum* under heat stress, accounting for 37.44% of the total gene set, and observed many unique genes (42% of the upregulated genes and 24% of the downregulated genes, respectively). (**Supplementary Table 9**). The acquisition of unique genes may reflect the unique and powerful evolutionary pressures that a species undergoes when adapting to new environments (Van Oss and Carvunis, 2019; Silva et al., 2021), and which differential expressions suggest that unique genes may have originated as a result of the drive for heat stress that *N. japonicum* once experienced (Arendsee et al., 2014). Additionally, we found physical clusters of many genes in the upregulated genes. This chromosomal clustering may confer a selective advantage through its ability to coordinate gene regulation at the chromatin level (Reimegård et al., 2017), and the physical clusters of upregulated genes may have a potential cell economy for *N. japonicum* under heat stress (Hurst et al., 2004; Pecrix et al., 2018). Such gene-organized co-expression appears to be common in eukaryotes and has been found in specific metabolic pathways in various plants (Nützmann et al., 2016), in *A. thaliana* stamen development-related genes (Reimegård et al., 2017), in symbiosis-related islands in *M. truncatula* (Pecrix et al., 2018), and in the gene components of *S. caninervis* in response to drought stress (Silva et al., 2021).

Using WGCNA, we investigated alterations in gene expression during different stages of high-temperature stress. Interestingly, we identified terms related to the response to antibiotics (**Figures 5A**). Since our experimental protocol did not include the administration of antibiotics, the significant enrichment of this term may be due to a certain degree of similarity in response between high-temperature stress and antibiotic stress (Cruz-Loya et al., 2019). The hub genes (122) of *N. japonicum* in response to heat stress were identified, with our focus on the M1 module, which was linked significantly with H1 due to its diversity of GO terms (**Supplementary Table 16**). Additionally, we analyzed the composition and potential functions of hub genes (**Supplementary Table 17**), such as *NJ13G012500* was homologous to the *ORP1B* of *A. thaliana* and may function as an endogenous and exogenous danger signaling molecule that triggers plant innate immunity in plants (Chen et al., 2022). *NJ02G004190* was homologous to *HBP2* and had properties suitable for tetrapyrrole carrier proteins. *NJ14G003380* encoded a chloroplast cyclophilin functioning in the assembly and maintenance of photosystem II (PSII) super complexes (Fu et al., 2007). In addition, *BAG6* (Echevarría-Zomeño et al., 2016) and *ROF2* (Meiri et al., 2010) may be involved in limiting the extension of the heat stress response in *A. thaliana*, with their homologs in the hub genes of *N. japonicum* (*NJ03G021340* and *NJ09G002390*, respectively). These putative hub genes may be favored for genetic transformation into crops. Surprisingly, 35.25% of hub genes are neighboring genes, and we suggest that the occurrence of these genes may be due to expression piggybacking (Dai et al., 2014; Ghanbarian and Hurst, 2015; Lan and Pritchard, 2016), which allows quick response and tight regulation during heat stress, at the lowest energy expense.

## Materials and methods

### Plant materials

Wild gametophyte of *N. japonicum* was collected from Qianshan, Anqing, Anhui Province, China. After collection, the material was further identified morphologically and the voucher specimen (collection number: DNA1220) had been deposited at the Fairy Lake Botanical Garden, Shenzhen & Chinese Academy of Sciences, Shenzhen, Guangdong Province, China. The fresh gametophyte sample of *N. japonicum* was cleaned with distilled water and dried using lab paper, then the plant tissues were examined under a dissecting microscope to avoid potential contaminations from other plants, and used for subsequent experiments.

### Heat stress experiments

A stress temperature (42°C) of four time gradients (cultured at 42°C for 1 h, 3 h, 6 h, and 12 h) and a control at 20°C were set up, with three biological replicates for each gradient. The *N. japonicum* samples from the four time gradients were first cultured at 35°C for 1 h to allow them to acclimate to the high temperature and to prevent them from entering dormancy due to sudden heat stress. They were then transferred to 42°C and three samples were taken at 1 h, 3 h, 6 h, and 12 h respectively, immediately treated with liquid nitrogen and stored in a −80°C freezer.

### DNA and RNA sequencing

Genomic DNA and RNA were extracted using FastPureTM Plant DNA Isolation Mini Kit (Vazyme, Nanjing, China) and RNA-easyTM Isolation Reagent (Vazyme, Nanjing, China), respectively. DNA and RNA quantification and qualification were performed using 1% agarose gel electrophoresis, a Qubit 2.0 fluorometer (Thermo Fisher Scientific, USA), and a NanoDrop 2000 spectrophotometer (Thermo Fisher Scientific, USA). Nanopore libraries were prepared using SQK-LSK108 and sequenced on a Nanopore PromethION sequencer. DNA libraries for short-read whole genome sequencing (WGS) were constructed using the Illumina TruSeq DNA PCR-free library preparation kit (Illumina, CA, USA) with 300- to 500-bp fragment sizes, and sequenced on the Illumina NovaSeq 6000 platform to generate 150-bp paired-end (PE) reads. Transcriptome libraries were constructed with a TruSeq RNA Library Prep Kit v2 (Illumina, CA, USA) with an insert size of 200–400 bp, after polyA selection, and sequenced on an Illumina NovaSeq 6000 platform, and 150-bp PE reads were generated. The Hi-C library construction process includes cross-linking, restricted enzyme digestion (MboI), end repair, DNA cyclization, and purification (Yu et al., 2022). PE-150-bp reads were generated on Illumina NovaSeq 6000 platforms.

### Genome size estimation

Low-quality reads and adapter sequences were filtered using Trimmomatic (v0.39) (Bolger et al., 2014). We then performed *K*-mer analyses to estimate the genome size of *N. japonicum* using clean Illumina reads. GCE (v1.0.2) (Liu et al., 2013) was used to calculate the *K*-mer distribution, and genome size was estimated by dividing the total number of *K*-mer by the *K*-mer peak depth. The haploid genome size of *N. japonicum* was estimated to be 184.22 Mb (**Supplementary Figure 2**).

### Genome assembly

The *de novo* assembly of the *N. japonicum* genome was performed using Nextdenovo v2.5.0 (https://github.com/Nextomics/NextDenovo) with default parameters and 79.16-Gb Nanopore long reads. The primary assembly was polished three times with Nanopore long reads using NextPolish v1.3.1 (https://nextpolish.readthedocs.io/en/latest/QSTART.html) to correct for structure variations and insertions/deletions followed by three rounds of single-nucleotide polymorphism and insertion/deletion correction using Pilon (v1.23) (Walker et al., 2014) with clean Illumina reads. To remove contaminating contigs, we first performed BLASTN search for the assembled contig sequences in the National Center for Biotechnology Information (NCBI) nucleotide collection (released in November 2022) with the following parameters: “-evalue 1e-5 -max_hsps 10000 -outfmt 6 -num_alignments 20000”; for each query sequence, the total number of unique coverage positions for all hits was calculated, and any query sequence with non-embryophyte coverage greater than 50% was considered a contaminating sequence and removed from the genome. Chloroplast and mitochondrial genomes were assembled using GetOrganelle (v1.7.5.0) (Jin et al., 2020) and NOVOPlasty (v3.5) (Dierckxsens et al., 2017) respectively, and then organelle fragments were removed using the same parameters. To validate the results of the decontamination procedure, we performed GC-depth analysis with a window size of 10 Kb (Yu et al., 2022). To further enhance assembly contiguity, Juicer (v1.6) (Durand et al., 2016b) was used to extract valid data from 103.05 Gb of Hi-C clean reads and 3D-DNA pipeline (Dudchenko et al., 2017) was employed to anchor, order, and orient the assembled scaffolds into 14 pseudochromosomes. Finally, Juicebox (v1.11.08) (Durand et al., 2016a) was used to manually adjust the results of the 3D-DNA pipeline.

### Repeat annotation

A combination of *de novo* and known repeat libraries was used to maximize the chances of identifying repetitive elements. The Piler (Edgar and Myers, 2005), LTR_FINDER (Xu and Wang, 2007), and RepeatScout (Price et al., 2005) were used to generate *de novo* repeat libraries. The Piler, LTR_FINDER, and RepeatScout repetitive libraries were combined and further used as the input data for RepeatMasker (Tarailo-Graovac and Chen, 2009). Repbase (v21.01) (Jurka et al., 2005) was a database of known repetitive elements that was searched using RepeatMasker and RepeatProteinMask (Tarailo-Graovac and Chen, 2009). Tandem repeats were identified using Tandem Repeats Finder (v4.07) (Benson, 1999).

### Gene annotation and functional annotation

BRAKER2 pipeline (v2.1.5) (Brůna et al., 2021) was used to predict PCGs based on the soft-masked *N. japonicum* genome. For details, proteome sequences of seven embryophytes (i.e., *A. thaliana*, *Azolla filiculoides*, *M. polymorpha*, *Oryza sativa*, *P. patens*, *Salvinia cucullata*, and *Selaginella moellendorffii*) obtained from the Phytozome v13 database (https://phytozome-next.jgi.doe.gov/) or Fernbase (https://fernbase.org/) were used to provide homology-based protein evidence, and transcriptome clean reads were mapped to the genomes using TopHat2 (v2.1.1) (Kim et al., 2013) to provide expressed sequence tag (EST) evidence. The completeness of genome assembly was assessed by the BUSCO (v3.1.0) (Simão et al., 2015) using the Viridiplantae odb10 set. For gene functional annotation, the annotated protein sequences were blasted against the UniProt (Swiss-Prot and TrEMBL) and TAIR databases using DIAMOND (v2.0.15) (Buchfink et al., 2015) with an E-value cutoff of <1 × 10^–5^. The GO annotation of gene models was carried out using eggNOG -mapper (v2) (Cantalapiedra et al., 2021), InterProScan (v5.51-85.0) (Jones et al., 2014), and PANNZER2 (Törönen et al., 2018). The KEGG annotation of gene models was performed using eggNOG-mapper and KofamKOALA (Aramaki et al., 2019). The domain of the gene models was identified by InterProScan. The plant TF prediction program iTAK online (v1.6) (Zheng et al., 2016) was used to identify TFs.

### Transcriptome assembly and mapping

Low-quality reads and adapters from the raw reads of transcriptome sequences were filtered using Trimmomatic. The resulting clean reads were *de novo* assembled using Trinity (v2.8.4) (Haas et al., 2013). For genes with more than one transcript, the longest transcript was chosen as the unigene. We merged the unigenes of 15 transcriptome samples and searched using BLASTN (E-value < 1 × 10^-5^) to remove non-embryophyte sequences. To extend the validation of genome assembly, the clean unigenes were compared to the reference assembly using BLASTN (E-value < 1 × 10^−10^), and 88.79% of the annotated genes of *N. japonicum* were successfully mapped to unigenes.

### Identification of whole-genome duplication, reconstruction of ancestral chromosomes, and inter-genomic synteny of mosses

A synteny analysis method and a *K*_s_-based age distribution approach as described previously (Lang et al., 2018; Li et al., 2018b) were used to identify the WGD events. The jcvi (v1.1.8) was employed for drawing the dot plot to show the relationship of intra-genomic collinear blocks with a cscore cutoff of 0.99. Owing to the diversity of factors affecting plant substitution rates, *K*_s_ estimates for events of the same absolute age may differ depending on the synonymous substitution rates in the lineages involved (Sensalari et al., 2022). To accurately estimate *K*_s_ distributions of WGD events for *N. japonicum* and *K*_s_ values for divergence events among species, ksrates (v1.1.3) (Sensalari et al., 2022) was used to generate adjusted mixed plots of *K*_s_ distributions by rescaling ortholog *K*_s_ estimates of species divergence times to the paralog *K*_s_ scale of *N. japonicum* with the following parameters: “collinearity = yes, max_number_outgroups = 4”, and the Newick tree ((((*C. purpureus*, *N. japonicum*), *P. patens*), (*S. fallax*, *S. magellanicum*)), *Takakia lepidozioides*) was used as the input phylogeny. The coding sequences (CDS) for all species used genomic data from the Phytozome v13 database, except for the *T. lepidozioides*, which used transcriptomic data from the One Thousand Plant Transcriptomes Initiative (One Thousand Plant Transcriptomes Initiative, 2019).

Since the ancestral karyotype of *C. purpureus* (Carey et al., 2021) was known from published moss genomes and had a closer phylogenetic relationship to *N. japonicum* (Liu et al., 2019b), it was chosen as the reference species for reconstructing the ancestral chromosome elements of *N. japonicum* using WGDI (v0.6.1) (Sun et al., 2022a).

The identification of synteny gene pairs among moss genomes was performed using jcvi. Pairwise synteny was assessed by syntenic percentage (synteny gene pairs/all-by-all comparison results filtered). The all-by-all comparison was performed with LAST (http://last.cbrc.jp/) in jcvi software and filtered tandem duplications and weak hits.

### Duplicated gene categorization and calculating *K*_a_, *K*_s_, and *K*_a_/*K*_s_ values

Based on the method used by Qiao et al. (2019) to identify duplicated genes in bryophytes (**Supplementary Note**), the duplicated genes were classified into five different categories: WGD, TD, PD, TRD, and DSD. TRD referred to the duplication of ancestral and novel loci, and the ancestral loci could be divided into two categories: intra-genomic synteny genes and inter-species synteny genes (Qiao et al., 2019). KaKs_Calculator (v2.0) (Wang et al., 2010) was used to calculate *K*_a_ and *K*_s_ values of duplicated gene pairs by implementing the model averaging (MA) method in ParaAT (v2.0) (Zhang et al., 2012). To account for the saturated substitutions at synonymous sites, the *K*_s_ values > 5.0 were excluded from further analysis (Vanneste et al., 2013; Li et al., 2016; Qiao et al., 2019).

### Inter-genomic divergence of mosses

The longest protein sequence of each gene in the 12 published bryophyte genomes (1 hornwort, 1 liverwort, and 10 mosses) was selected for clustering using OrthoFinder (v2.3.11) (Emms and Kelly, 2019), from which *A. angustus* and *M. polymorpha* were selected as outgroups (**Supplementary Table 1**). A total of 69 single-copy orthologous genes were aligned using MAFFT (v7.453) (Katoh and Standley, 2013) and a maximum likelihood (ML) tree was constructed using IQ-TREE2 (v2.0.6) (Minh et al., 2020) with ultrafast 1,000 bootstrap replicates based on the JTT model. BEAST2 (v2.6.4) (Bouckaert et al., 2014) was used to estimate divergence times. The following fossil calibrations were used as priors: divergence of hornworts and liverworts, liverworts and mosses at approximately 407–515 Mya (Guo et al., 2012).

### Transcriptome analysis

Raw transcriptome sequencing data were filtered using Trimmomatic and then mapped to the reference genome using HISAT2 (v2.2.0) (Kim et al., 2019), and count and TPM values were calculated using the StringTie (v2.2.1) program (Pertea et al., 2015). In addition, genes with expression fold change > 2 and *p* < 0.05 were identified as DEGs using DESeq2 (Love et al., 2014) based on count values. To identify co-expressed genes during heat stress, WGCNA (Langfelder and Horvath, 2008) was used based on the genes with average TPM > 2 across 15 samples. To better visualize the expression levels of co-expressed module genes using ComplexHeatmap (Gu et al., 2016), the TPM data were normalized (*z*-score). For module genes that showed high correlation with a particular stage, we performed GO enrichment analyses using clusterProfiler (Yu et al., 2012). We followed the workflow of Pecrix et al. (2018) to locate physical clusters or “islands” of upregulated genes. Briefly, a genomic region was considered an “island” if ≥3 upregulated genes represented >60% of the expressed genes in a 50-Kb window.

### Identification of gene family

Gene family members were identified as follows. We first downloaded protein sequences of Self-incomp_S1, HSP, LEA, and PPR gene families from TAIR (https://www.arabidopsis.org/). Relevant gene families were searched from the gene set using BLASTP with an E-value cutoff of <1 × 10^–5^. The resulting sequences were annotated with Pfam protein domains using InterProScan, and the genes without the corresponding domains were removed. Finally, we performed alignments of the HSP and LEA gene families using MAFFT, respectively, and constructed ML trees using IQ-TREE2 with the JTT model based on ultrafast 1,000 bootstrap replicates.

## Data availability statement

The datasets presented in this study can be found in online repositories. The names of the repository/repositories and accession number(s) can be found below: National Genomics Data Center (NGDC; https://ngdc.cncb.ac.cn/bioproject/) under the the BioProject accession number PRJCA017860 and figshare (https://figshare.com/) data repository (doi: 10.6084/m9.figshare.23573514).

## Author contributions

XZ: Formal Analysis, Software, Visualization, Writing – original draft. TP: Formal Analysis, Visualization, Writing – original draft. YZ: Software, Writing – original draft. YC: Software, Writing – original draft. QZ: Writing – review & editing. LZ: Writing – review & editing. SD: Funding acquisition, Project administration, Writing – review & editing. YL: Funding acquisition, Project administration, Writing – review & editing.
