# Supplementary material for: Chromosome-level genome assembly of Niphotrichum japonicum provides new insights into heat stress responses in mosses
Source: Front Plant Sci. 2023 Oct 18;14:1271357. doi: 10.3389/fpls.2023.1271357 (PMC10619864; doi:10.3389/fpls.2023.1271357)
Supplement: Supplementary file 1 [file DataSheet_1.docx]

***Supplementary Figures***

**Chromosome-level genome assembly of *Niphotrichum japonicum* provides new insights into heat stress responses in mosses**

Xuping Zhou^1,2,†^, Tao Peng^2,†^, Yuying Zeng^3,4^, Yuqing Cai^3,4^, Qin Zuo^1^, Li Zhang^1^, Shanshan Dong^1,*^, Yang Liu^1,3,*^

^1^Laboratory of Southern Subtropical Plant Diversity, Fairy Lake Botanical Garden, Shenzhen & Chinese Academy of Sciences, Shenzhen, China.

^2^Colleage of Life Sciences, Guizhou Normal University, Guiyang, China.

^3^State Key Laboratory of Agricultural Genomics, BGI Research, Shenzhen, China.

^4^College of Life Sciences, University of Chinese Academy of Sciences, Beijing, China.

*** Correspondence:**

Correspondence author

Shanshan Dong

E-mail: shangrilass@163.com

Yang Liu

E-mail: yang.liu0508@gmail.com

**Supplementary Figure 1.** The morphology of *N. japonicum* gametophytes.


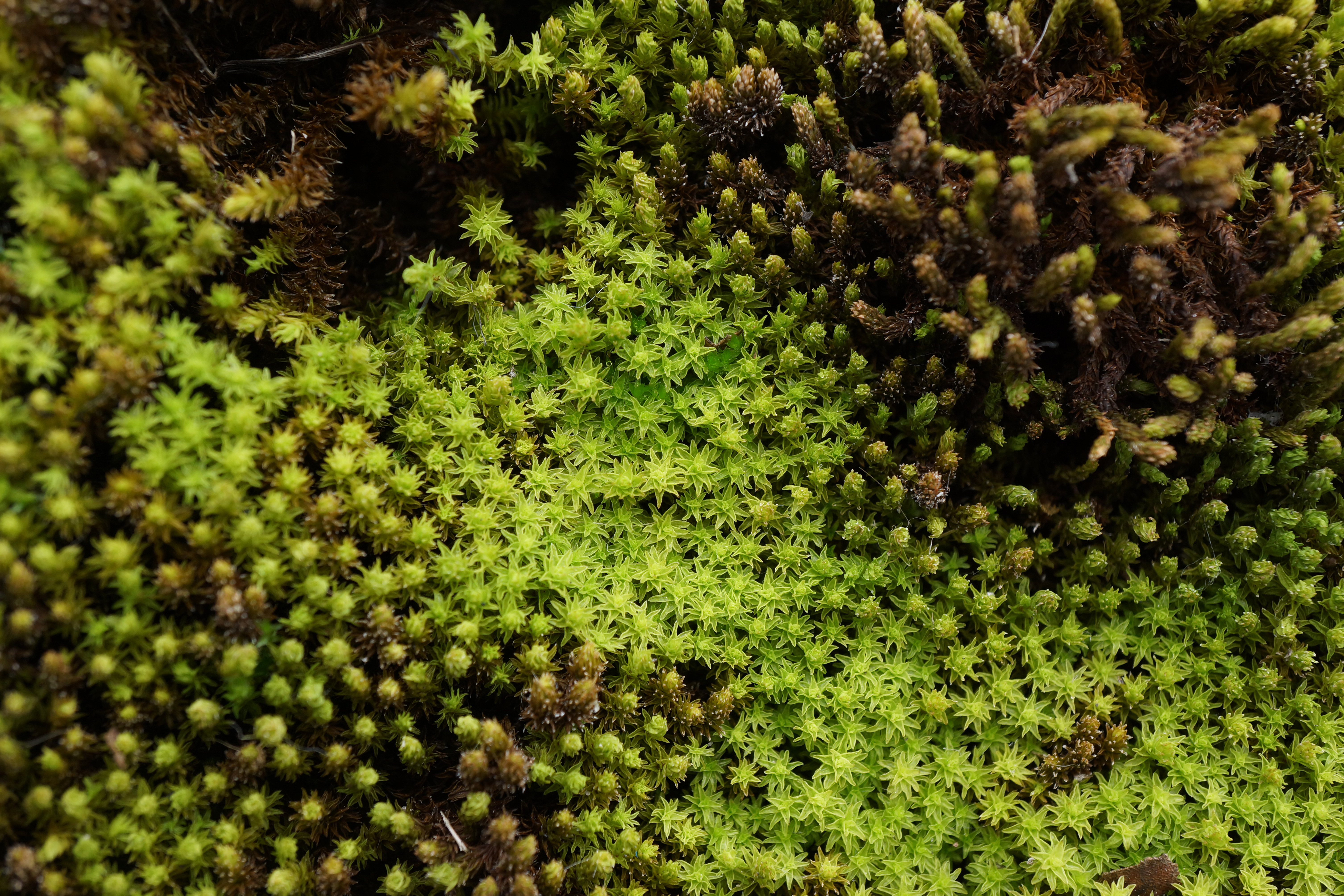


**
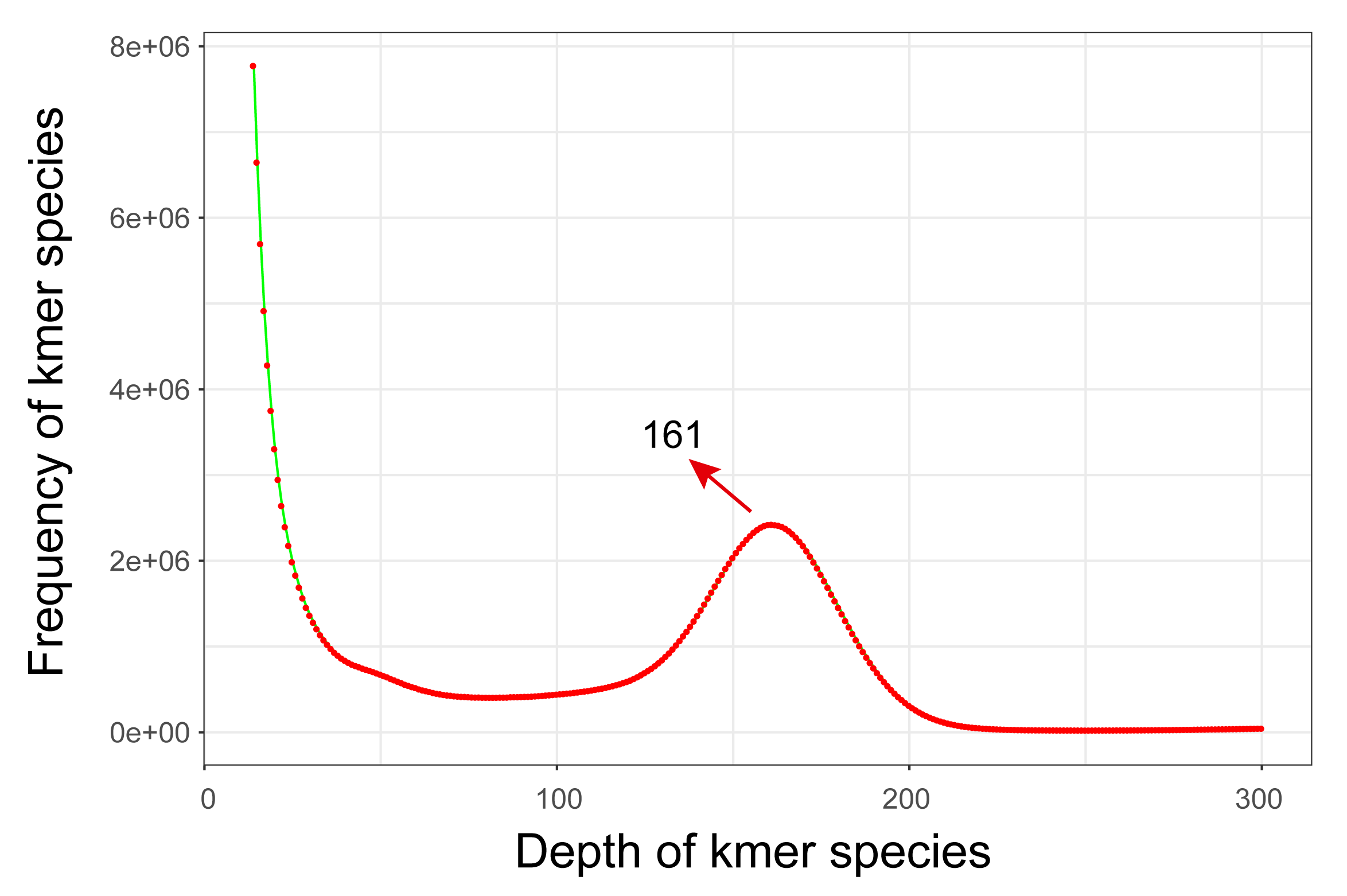
 Supplementary Figure 2.** *K*-mer distribution (K=19) of Illumina sequencing reads.


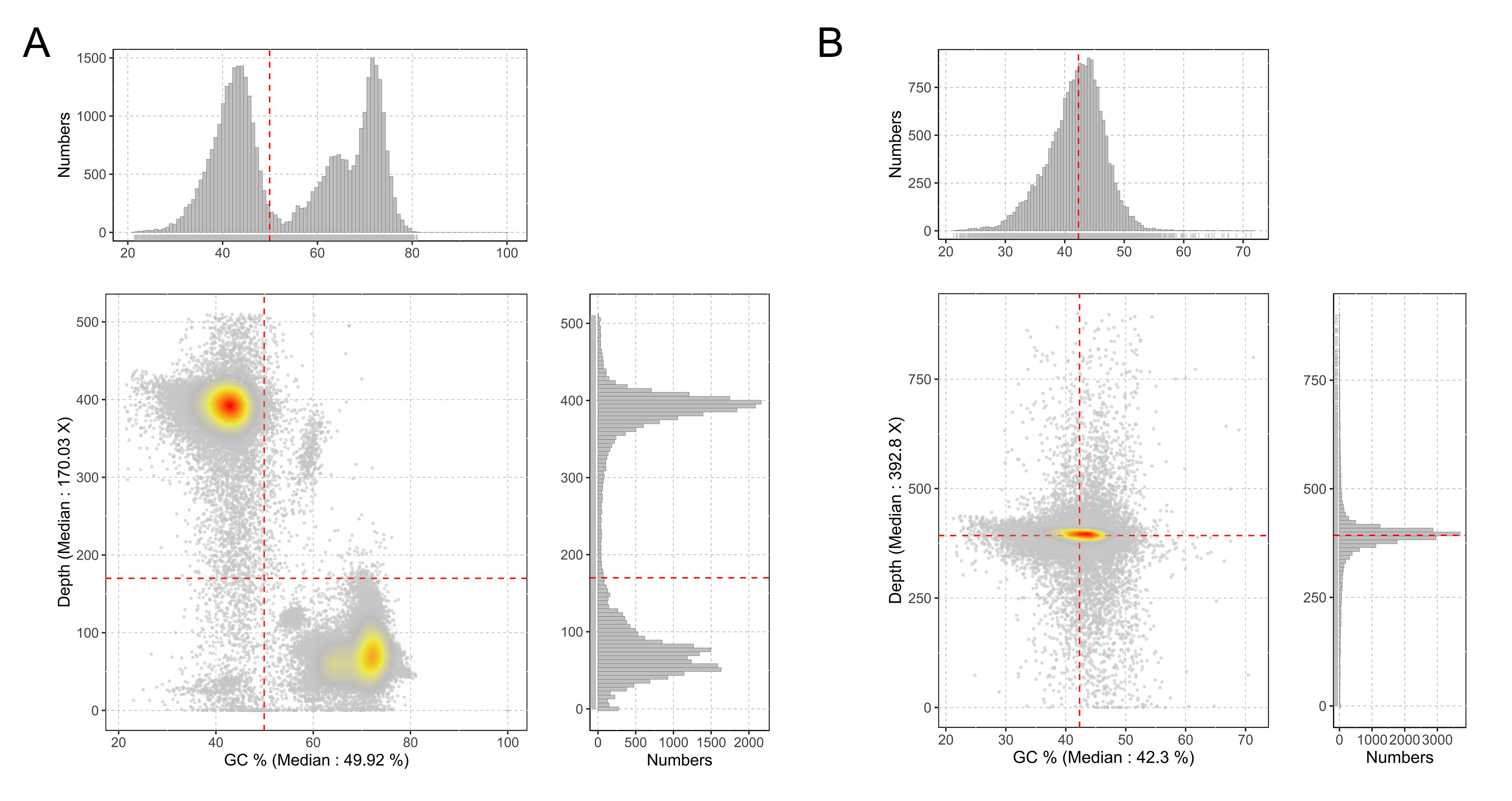


**Supplementary Figure 3.** Comparison of GC content and sequencing depth of the *N. japonicum* genome before and after decontamination. **(A)** Before decontamination. **(B)** After decontamination.

**
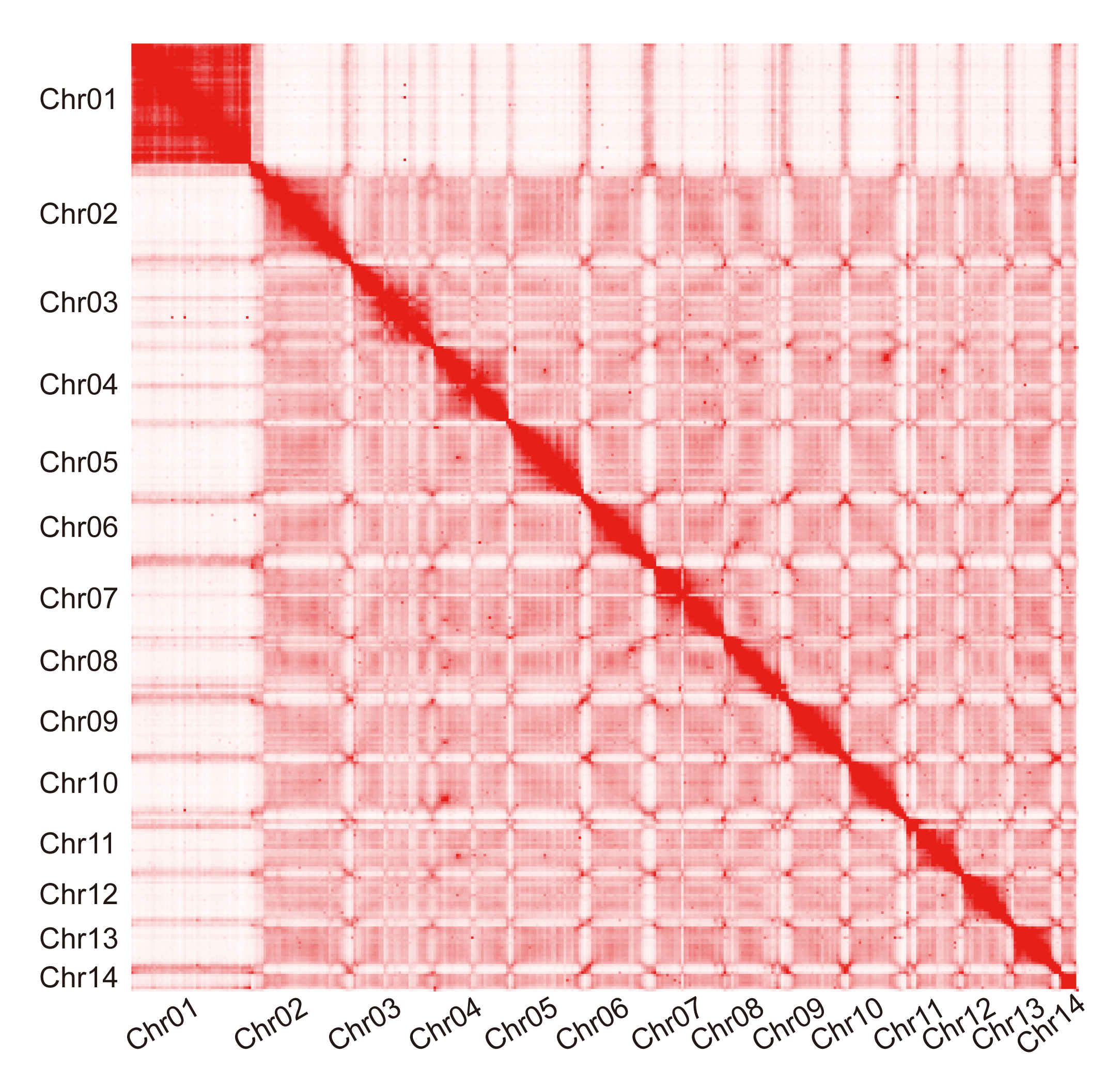
**

**Supplementary Figure 4.** Genome-wide chromatin interactions of 14 chromosomes in *N. japonicum*.

**
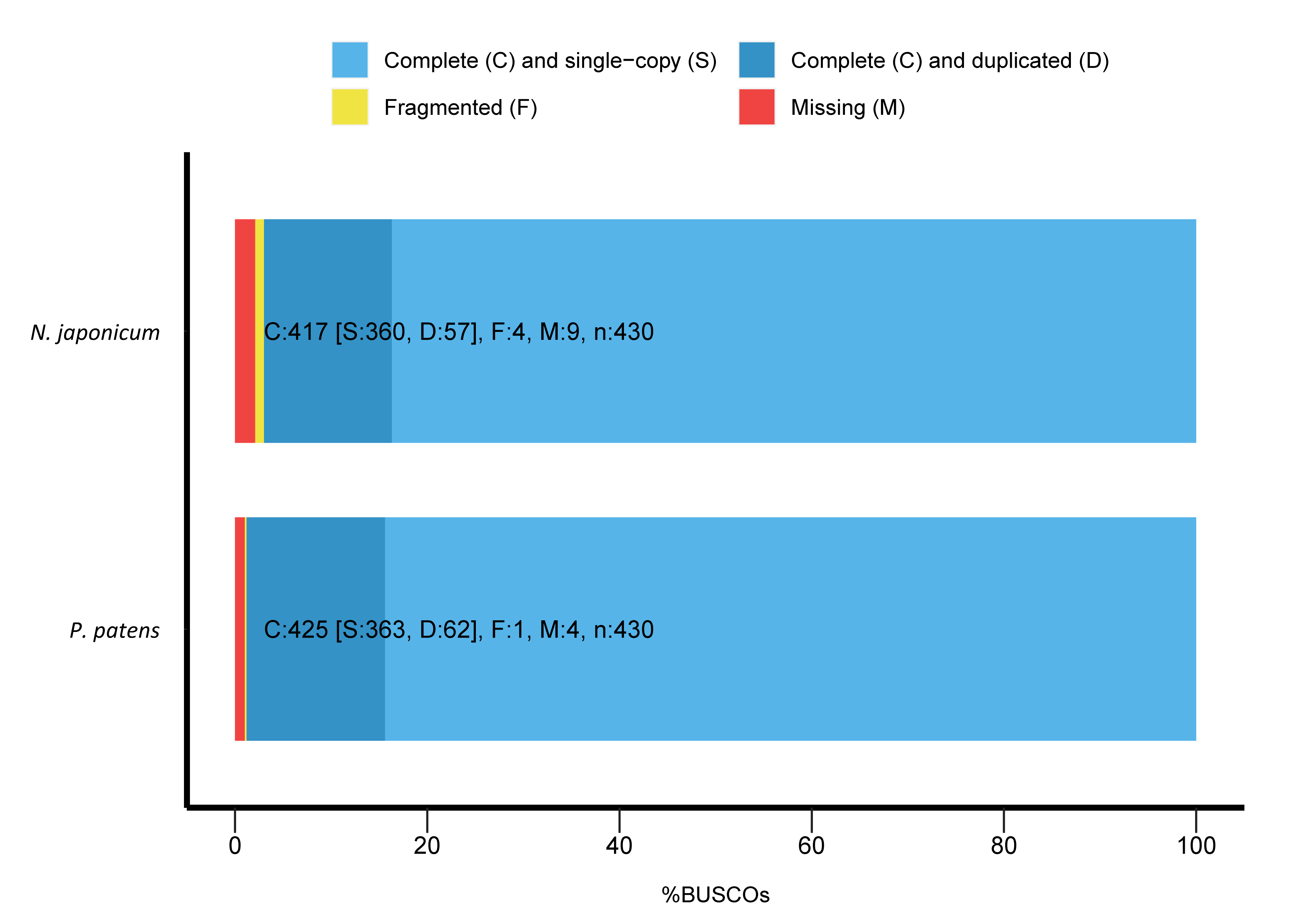
Supplementary Figure 5.** BUSCO analysis of *N. japonicum* and *P. patens* v3.3 genomes.


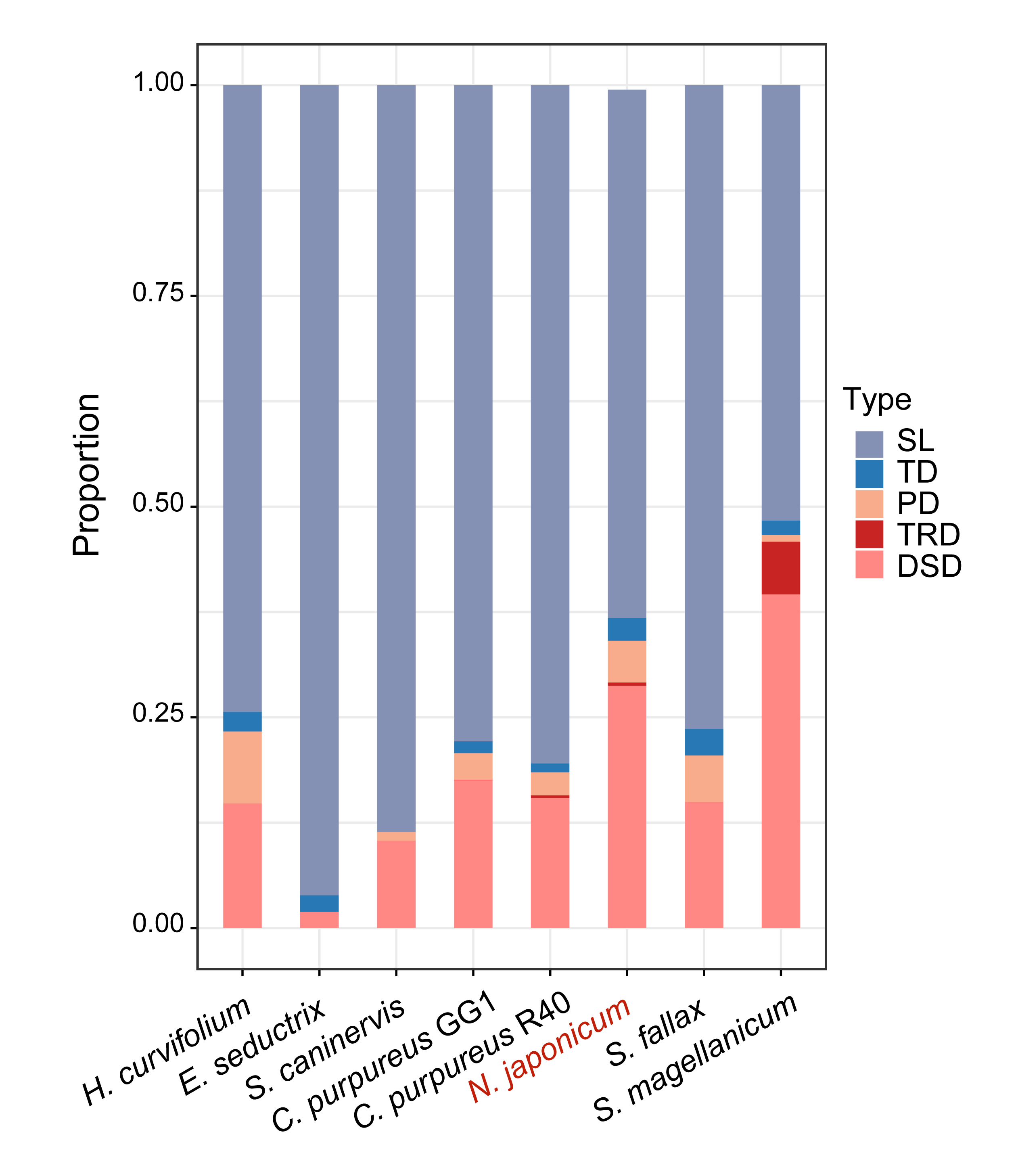


**Supplementary Figure 6.** Composition of duplicated genes on the sex chromosomes of mosses, SL: Singleton, TD: tandem duplication, PD: proximal duplication, TRD: transposed duplication, DSD: dispersed duplication.


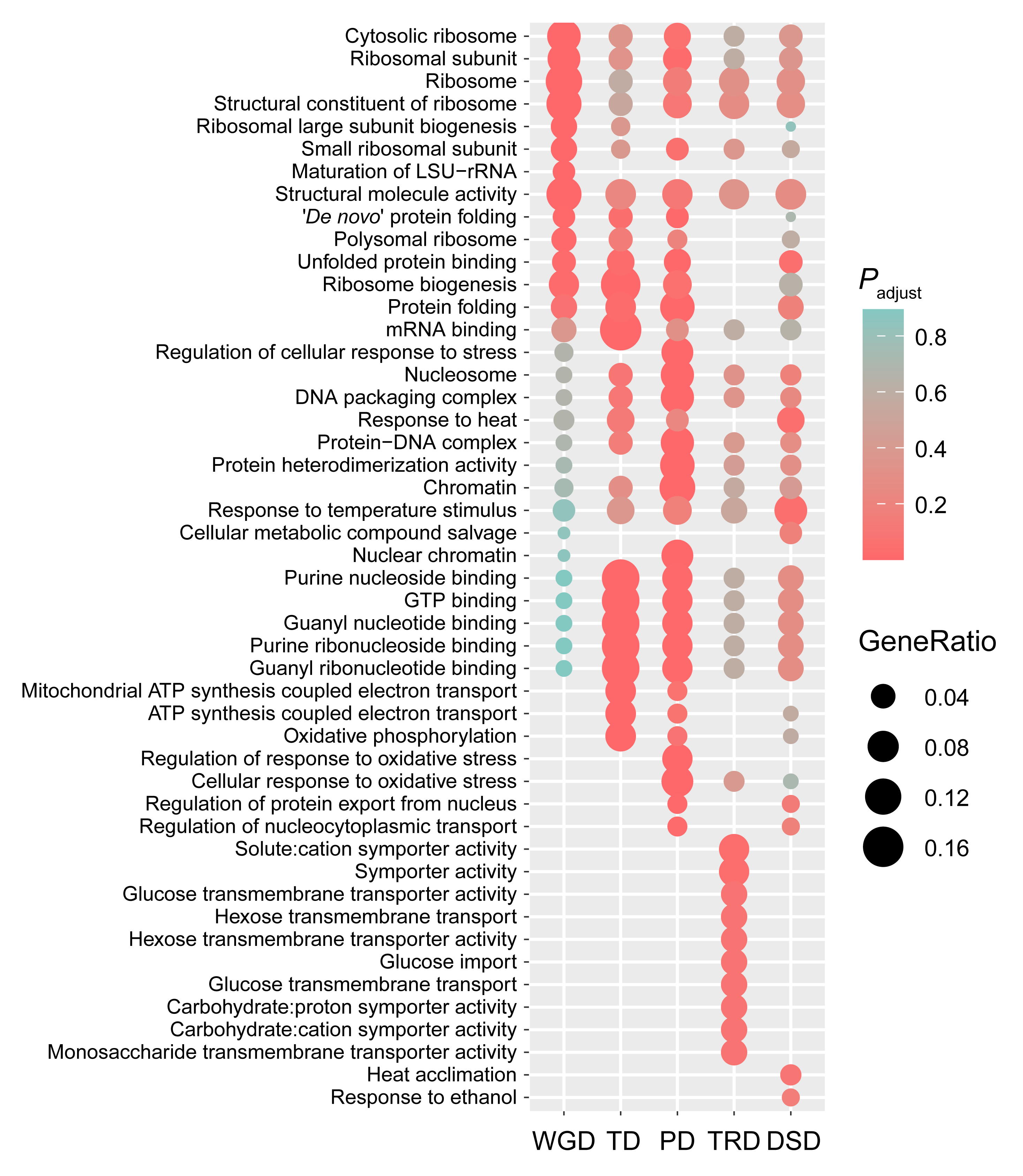


**Supplementary Figure 7.** GO enrichment analysis of up-regulated genes in different duplication modes of *N. japonicum*. All genes of *N japonicum* annotated by GO library as background information, and the top 10 GO terms of each duplicated mode were selected for display based on *P*_adjust._

**
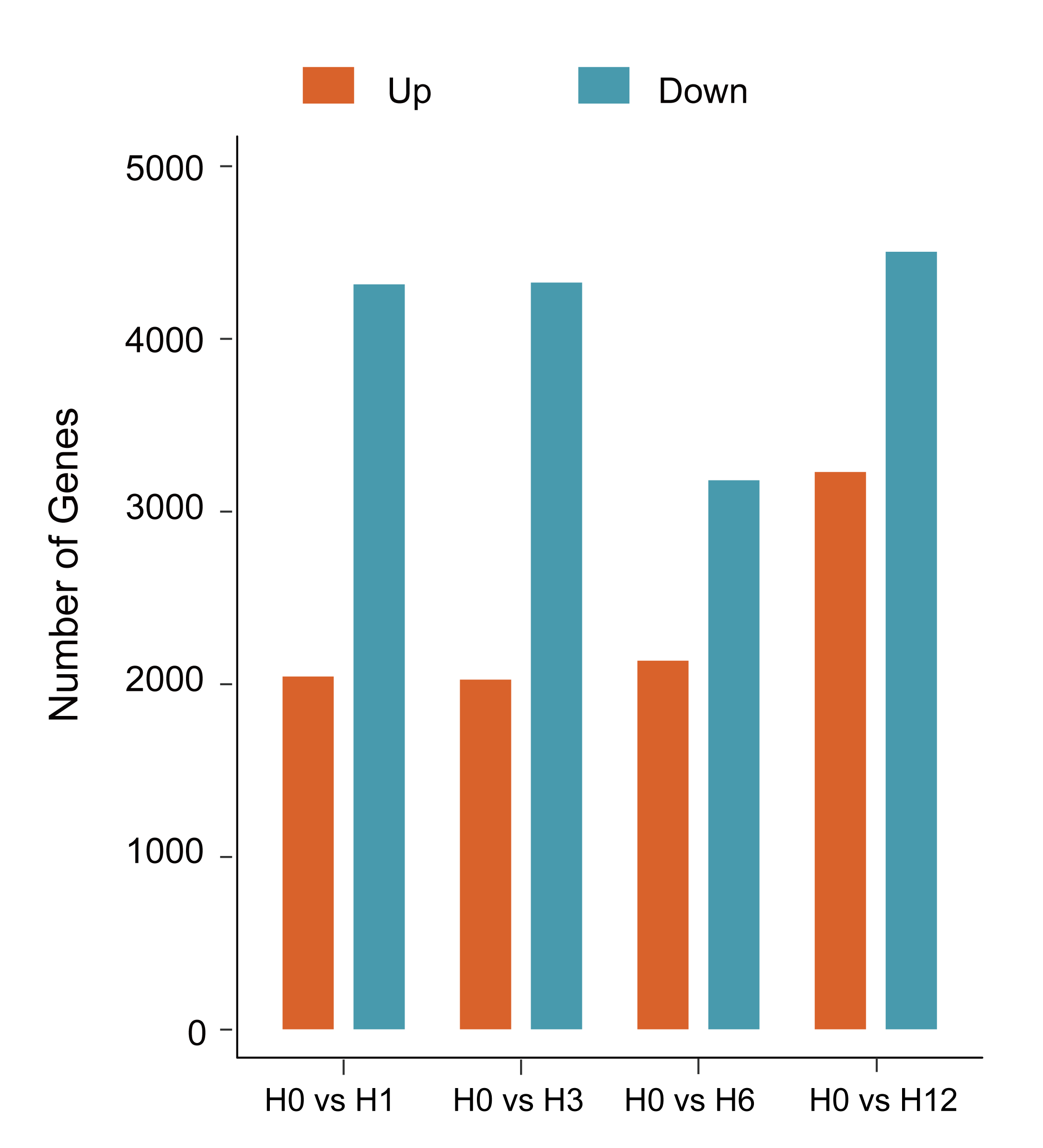
**

**Supplementary Figure 8.** Statistics of differentially expressed genes (DEGs) at 1 h, 3 h, 6 h and 12 h of heat stress.


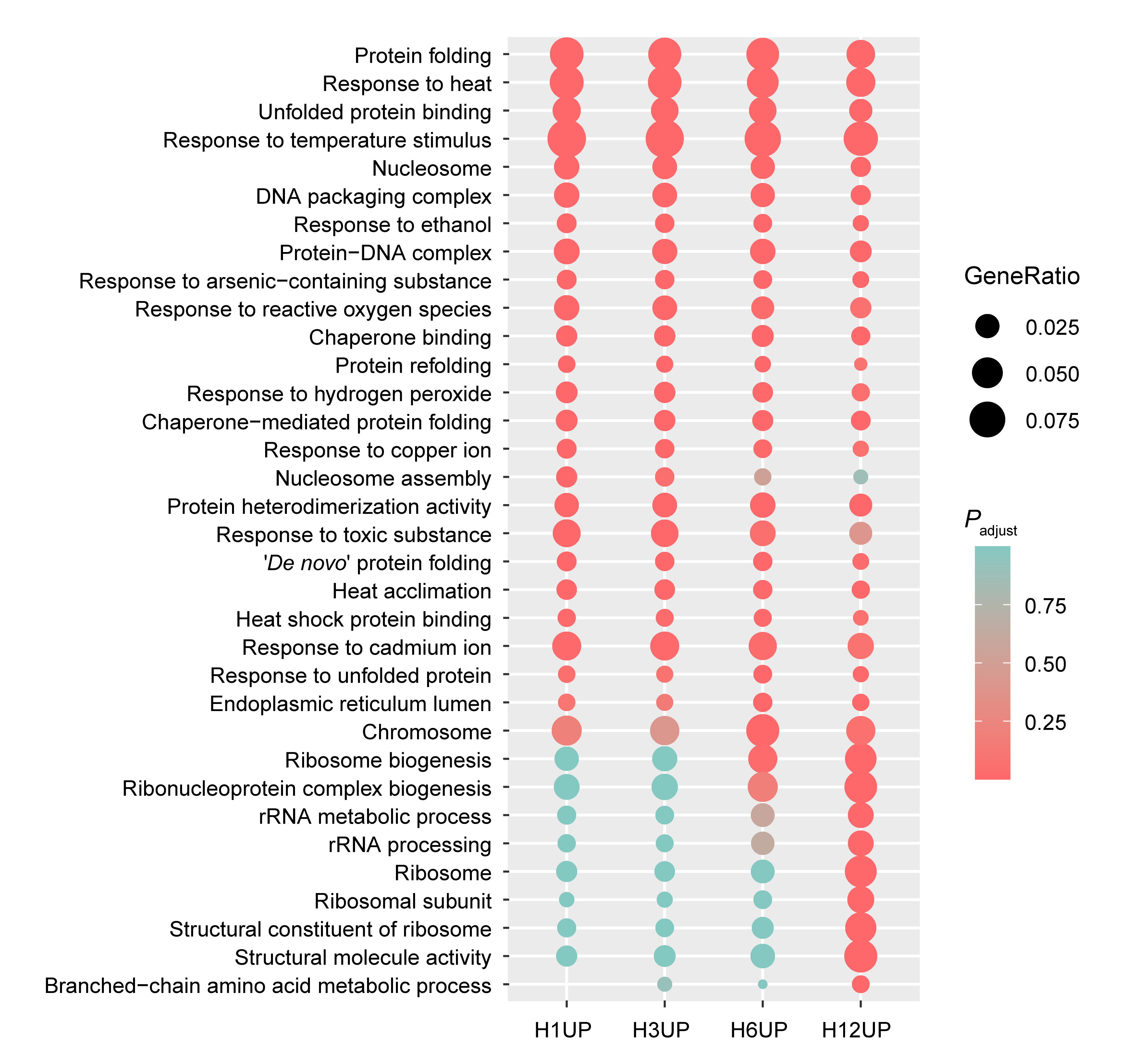


**Supplementary Figure 9.** GO enrichment analysis of up-regulated genes in four stages of heat treatment. H1UP, H3UP, H6UP, H12UP represent genes up-regulated by H1, H3, H6, and H12 versus the control samples (H0), respectively. All genes of *N japonicum* annotated by GO library as background information, and the top 20 GO terms of four stages were selected for display based on *P*_adjust._

**
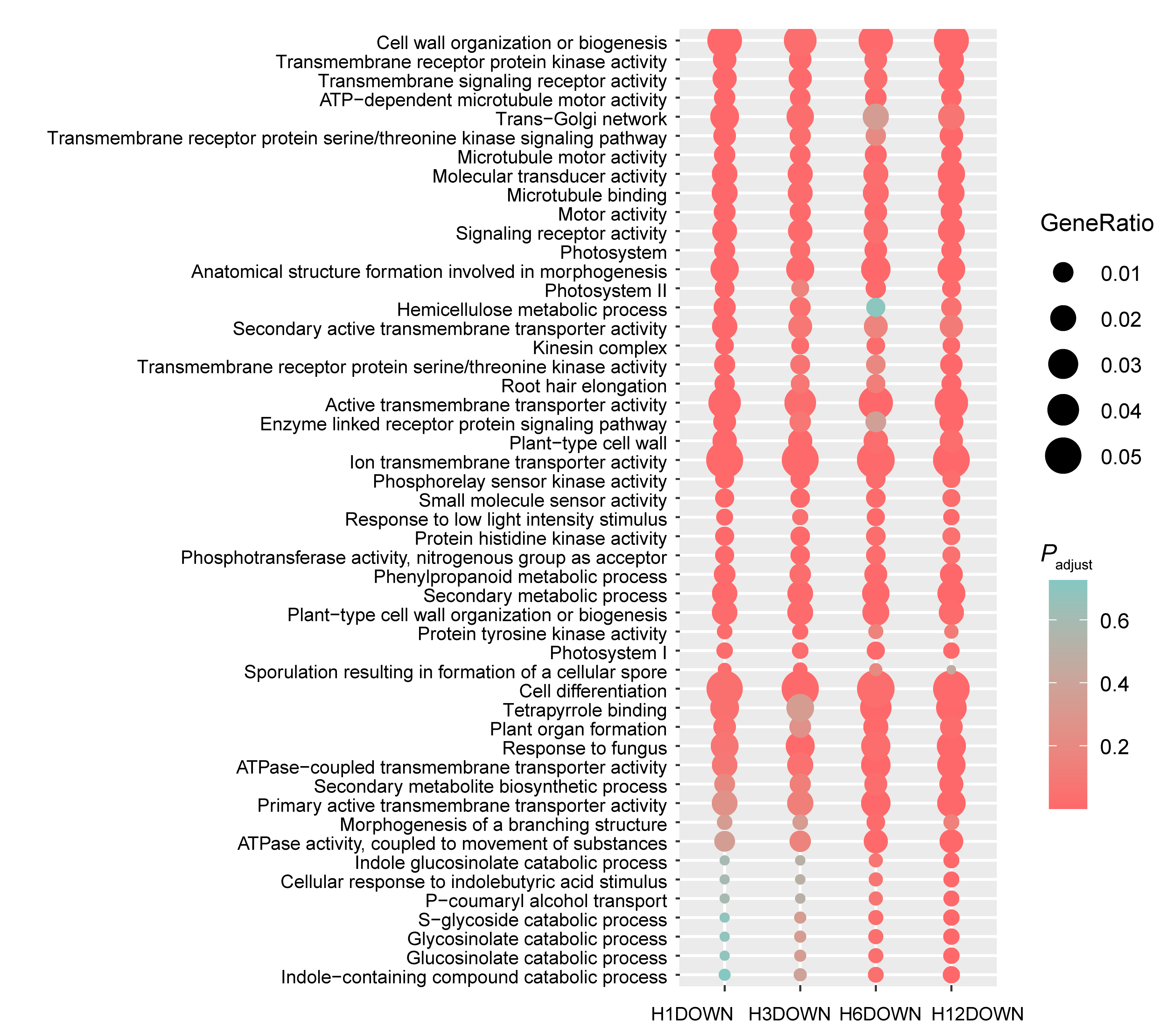
Supplementary Figure 10.** GO enrichment analysis of down-regulated genes in four stages of heat treatment. H1DOWN, H3DOWN, H6DOWN, H12DOWN represent genes downregulated by H1, H3, H6, and H12 versus the control samples (H0), respectively. All genes of *N japonicum* annotated by GO library as background information, and the top 20 GO terms of four stages were selected for display based on *P*_adjust._


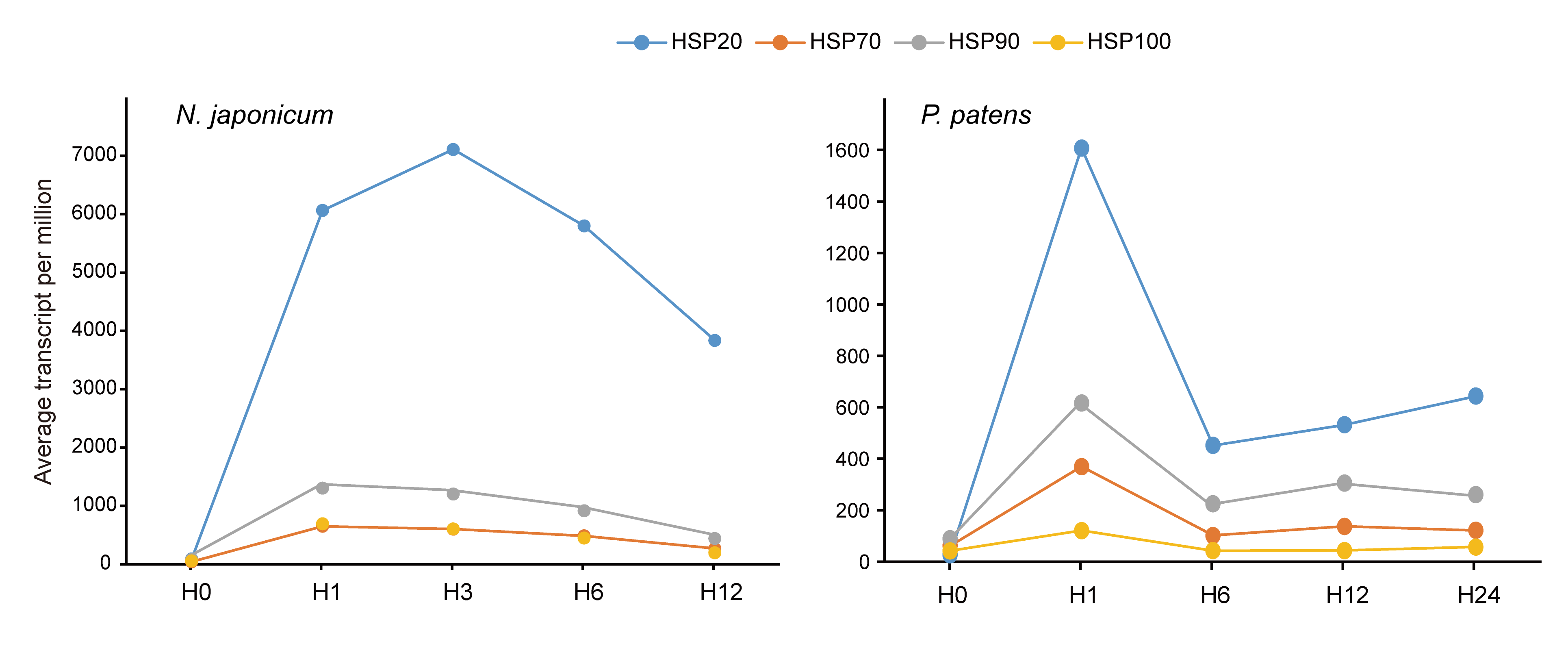


**Supplementary Figure 11.** Expression patterns of different HSPs in *N. japonicum* and *P. patens* under heat stress. For *P. patens*, heat treatment transcriptome data were downloaded from NCBI (accession: PRJNA576004). H0 represents 1 h at 24°C, and H1, H6, H12, and H24 represent 1, 6, 12, and 24 h of treatment at 37°C, respectively.





**Supplementary Figure 12.** Phylogeny of the LEAs in *N. japonicum*, *P. patens* and *A. thaliana*. SMP, seed maturation protein. The numbers in brackets represent the number of subfamilies in *N. japonicum*, *P. patens* and *A. thaliana*, respectively.
