# Supplementary material for: Chromosome-level genome assembly of Niphotrichum japonicum provides new insights into heat stress responses in mosses
Source: Front Plant Sci. 2023 Oct 18;14:1271357. doi: 10.3389/fpls.2023.1271357 (PMC10619864; doi:10.3389/fpls.2023.1271357)
Supplement: Supplementary file 2 [file DataSheet_2.docx]

***Supplementary Note***

**Chromosome-level genome assembly of *Niphotrichum japonicum* provides new insights into heat stress responses in mosses**

Xuping Zhou^1,2,†^, Tao Peng^2,†^, Yuying Zeng^3,4^, Yuqing Cai^3,4^, Qin Zuo^1^, Li Zhang^1^, Shanshan Dong^1,*^, Yang Liu^1,3,*^

^1^Laboratory of Southern Subtropical Plant Diversity, Fairy Lake Botanical Garden, Shenzhen & Chinese Academy of Sciences, Shenzhen, China.

^2^Colleage of Life Sciences, Guizhou Normal University, Guiyang, China

^3^State Key Laboratory of Agricultural Genomics, BGI Research, Shenzhen, China.

^4^College of Life Sciences, University of Chinese Academy of Sciences, Beijing, China.

*** Correspondence:**

Correspondence author

Shanshan Dong

E-mail: shangrilass@163.com

Yang Liu

E-mail: yang.liu0508@gmail.com

**Supplementary Note.** Identification of duplicated gene.

The duplicated genes were classified into five different categories (Qiao et al., 2019): WGD duplicates, tandem duplicates (TD), proximal duplicates (PD), transposed duplicates (TRD) and dispersed duplicates (DSD). The DupGen_finder (Qiao et al., 2019) was primarily used to identify duplicated genes. Studies had shown the presence of intra-genomic synteny in *Syntrichia caninervis* (Silva et al., 2021), but the number of syntenic blocks identified by DupGen_finder was 0. Additionally, TRD referred to the duplication of ancestral and novel loci, and the ancestral loci can be divided into two categories: intra-genomic synteny genes and inter-genomic synteny genes. Intra-genomic synteny genes were derived from WGD, so the identification of WGD will affected TRD. Based on this, the following steps were developed: a) DupGen_finder was used to classify duplicated genes, selecting the strict mode (unique), i.e., a gene only appears in one duplicated type in the final result, and increasing the parameter "-s" until WGD and intra-genomic synteny genes were not identified as ancestral loci for TRD. b) jcvi (Tang et al., 2008) was used to obtain WGD genes as intra-genomic synteny genes. c) As mosses had an ancient origin, identification using jcvi could result in the loss of many inter-genomic synteny genes (Qiao et al., 2019). Therefore, OrthoFinder (v2.3.11) (Emms and Kelly, 2019) was used to cluster the longest protein sequences from 11 mosses, *Marchantia polymorpha*, and *Anthoceros angustus*. If a gene in the focal species genome had orthologs in at least two other species, it was considered ancient and could exist in the common ancestor of bryophytes as an inter-genomic synteny genes. d) Based on the priority of duplication modes, i.e., WGD>TD>PD>TRD>DSD (Qiao et al., 2019), for TD and PD identified by DupGen_finder that could contain WGD or TRD, use intra-genomic synteny genes to search. If a gene pair matches with intra-genomic synteny genes, it was identified as a WGD gene pair and is removed. For DSD, the specific steps were: (Ⅰ) Using intra-genomic synteny genes to search first. If a pair of genes could match with intra-genomic synteny genes, it was removed, resulting in a gene set without WGD gene pairs. (Ⅱ) Combined the intra-genomic synteny genes and the inter-genomic synteny genes to obtain the ancestral gene set. If a gene pair in the WGD-free gene set obtained in (Ⅰ) matches with the ancestral gene set, it was retained as DSD gene pairs. If only one gene of pairs matched with the ancestral gene set, it was identified as a TRD gene pair. If neither gene of pair matched with the ancestral gene set, it was retained as DSD gene pairs. (e) The final WGD, TD, PD, TRD, DSD were obtained by deleting the genes present in the previous level according to the previously priority of the duplicated modes.

**References**

Emms, D.M., and Kelly, S. (2019). OrthoFinder: phylogenetic orthology inference for comparative genomics. *Genome Biol.* 20**,** 238. doi: 10.1186/s13059-019-1832-y.

Qiao, X., Li, Q., Yin, H., Qi, K., Li, L., Wang, R., et al. (2019). Gene duplication and evolution in recurring polyploidization-diploidization cycles in plants. *Genome Biol.* 20**,** 38. doi: 10.1186/s13059-019-1650-2.

Silva, A.T., Gao, B., Fisher, K.M., Mishler, B.D., Ekwealor, J.T.B., Stark, L.R., et al. (2021). To dry perchance to live: Insights from the genome of the desiccation-tolerant biocrust moss *Syntrichia caninervis*. *Plant J.* 105**,** 1339-1356. doi: 10.1111/tpj.15116.

Tang, H., Bowers, J.E., Wang, X., Ming, R., Alam, M., and Paterson, A.H. (2008). Synteny and Collinearity in Plant Genomes. *Science* 320**,** 486-488. doi:10.1126/science.1153917.
